# A statistical boosting framework for polygenic risk scores based on large-scale genotype data

**DOI:** 10.3389/fgene.2022.1076440

**Published:** 2023-01-10

**Authors:** Hannah Klinkhammer, Christian Staerk, Carlo Maj, Peter Michael Krawitz, Andreas Mayr

**Affiliations:** ^1^ Institute for Medical Biometry, Informatics and Epidemiology, Medical Faculty, University of Bonn, Bonn, Germany; ^2^ Institute for Genomic Statistics and Bioinformatics, Medical Faculty, University of Bonn, Bonn, Germany; ^3^ Center for Human Genetics, University of Marburg, Marburg, Germany

**Keywords:** polygenic risk score (PRS), high-dimensional data, variable selection, boosting, GWAS—genome-wide association study, prediction

## Abstract

Polygenic risk scores (PRS) evaluate the individual genetic liability to a certain trait and are expected to play an increasingly important role in clinical risk stratification. Most often, PRS are estimated based on summary statistics of univariate effects derived from genome-wide association studies. To improve the predictive performance of PRS, it is desirable to fit multivariable models directly on the genetic data. Due to the large and high-dimensional data, a direct application of existing methods is often not feasible and new efficient algorithms are required to overcome the computational burden regarding efficiency and memory demands. We develop an adapted component-wise *L*_2_-boosting algorithm to fit genotype data from large cohort studies to continuous outcomes using linear base-learners for the genetic variants. Similar to the snpnet approach implementing lasso regression, the proposed snpboost approach iteratively works on smaller batches of variants. By restricting the set of possible base-learners in each boosting step to variants most correlated with the residuals from previous iterations, the computational efficiency can be substantially increased without losing prediction accuracy. Furthermore, for large-scale data based on various traits from the UK Biobank we show that our method yields competitive prediction accuracy and computational efficiency compared to the snpnet approach and further commonly used methods. Due to the modular structure of boosting, our framework can be further extended to construct PRS for different outcome data and effect types—we illustrate this for the prediction of binary traits.

## 1 Introduction

In times of next-generation sequencing and decreasing costs for whole genome sequencing, the amount of available genotype data has increased dramatically in recent years, giving rise to new genetic insights (Beesley et al., 2020; National Human Genome Research Institute, 2021).

Polygenic risk scores (PRS) measure the individual genetic liability to a certain trait and can provide relevant information in the context of disease-risk stratification. In contrast to high-impact monogenic variants, which are mostly rare and have a high effect size, PRS are derived from common variants such as single-nucleotide polymorphisms (SNPs) with low or medium effect sizes. Polygenic effects could also explain part of the incomplete penetrance seen in many identified monogenic variants, as for example in the genes BRCA1 and BRCA2 both leading to a highly increased risk of breast cancer (Kuchenbaecker et al., 2017). Recent studies on the UK Biobank suggest that high-impact monogenic variants, PRS and family history could contribute additively to the risk of developing breast and prostate cancer (Hassanin et al., 2021). Despite these findings, PRS still lack to explain relevant parts of the estimated heritability of many traits.

PRS are typically derived as a sum of risk allele counts weighted by univariate effect estimates of the measured variants based on summary statistics from genome-wide association studies (GWAS) (Choi et al., 2020). Despite several approaches to account for linkage disequilibrium (LD, referring to the correlation structure between variants) and for the selection of informative variants (Euesden et al., 2014; Vilhjálmsson et al., 2015; Mak et al., 2017; Privé et al., 2021), the univariate structure of the estimation cannot fully account for interdependencies between the variants. For example, lassosum (Mak et al., 2017) adopts an *L*_1_ penalty term and solves a lasso-like problem while only using summary statistics and a LD reference panel. However, as published summary statistics and LD reference panels are most often based on different samples, lassosum can generally only approximate the full lasso path. A natural extension of using effect estimates from univariate models could hence be to fit a single multivariable model. While this approach seems natural from a methodological perspective, a direct application of existing methods is typically infeasible due to the high dimensionality of the genotype data, which can easily exceed the available computer memory. Recently, some approaches have been proposed to overcome this computational burden (Privé et al., 2018; Qian et al., 2020; Maj et al., 2022). In particular, Qian et al. proposed the so-called batch screening iterative lasso (BASIL) algorithm to fit the lasso on the complete original genotype data (Tibshirani, 1996; Qian et al., 2020; Li et al., 2022). The algorithm works on subsets of variants and computes the complete lasso path in an iterative fashion. Apart from the lasso, the algorithm can also be extended to other penalized regression methods such as the relaxed lasso (Meinshausen, 2007) or the elastic net (Zou and Hastie, 2005). In this context, Qian et al. were able to demonstrate that multivariable regularized PRS models fitted *via* the BASIL algorithm outperform the classical GWAS-based PRS for various traits such as height and high cholesterol.

While penalized regression models like the lasso and the elastic net impose explicit regularization, statistical boosting represents an alternative approach by introducing an implicit algorithmic regularization when combined with early stopping (Bühlmann and Hothorn, 2007; Mayr and Hofner, 2018). Boosting algorithms iteratively fit pre-defined base-learners to the gradient of the loss function, selecting the most influential base-learner in each step. The main tuning parameter of boosting algorithms is the number of iterations, which enables implicit variable selection and leads to sparse models. Due to its modular structure, boosting allows to combine possible base-learners with any convex loss function. These algorithms hence offer a great flexibility for statistical modelling, including various response types and the estimation of non-linear or other types of effects. A recent work has incorporated boosting into PRS modelling *via* a three-step approach (Maj et al., 2022): First, a marginal screening approach was applied on all variants to identify potentially informative ones. Then, multivariable algorithms including probing with boosting (Thomas et al., 2017) were applied on blocks of variants in LD to select (“fine-map”) the most informative variants. Finally, a statistical boosting model was fitted on the variant set created by joining the selected variants of all chunks. This approach yielded particularly sparse and interpretable models, whose predictive performance was superior to PRS derived by univariate methods like clumping and thresholding (Euesden et al., 2014) and was outperformed by the predictive performance achieved by the lasso *via* the BASIL algorithm. However this approach includes pre-filtering of the variants and is computationally demanding.

In this article we introduce a new framework to boost PRS, starting with a new computational approach to build *L*_2_-boosting models on large-scale genotype data for quantitative traits. Similar to the snpnet approach for the lasso, our algorithm iteratively works on smaller batches of variants. Yet, in contrast to recent boosting methods (Staerk and Mayr, 2021; Maj et al., 2022), the variants do not need to be pre-filtered in our snpboost approach and the batches are not pre-defined or randomly sampled, but chosen iteratively and deterministically in a data-driven way based on the correlations of the variants to the remaining residuals. By restricting the set of available base-learners in each step to those variants which were most correlated with residuals from a previous iteration, we are able to reduce the search space and decrease the computational time compared to a classical component-wise boosting algorithm.

We conducted a simulation study to examine the performance of our adapted boosting algorithm snpboost compared to the original *L*_2_-boosting on a reduced but still high-dimensional data set, on which the application of standard *L*_2_-boosting was still computationally feasible. Furthermore, we simulated data of higher dimensionality and larger sample size to investigate the influence of various hyperparameters (including the batch size) on the prediction accuracy and computational burden of the snpboost approach in a typical large-scale setting. We discuss reasonable default values for the hyperparameters which are incorporated in the provided R implementation (https://github.com/hklinkhammer/snpboost). Finally, we constructed multivariable PRS for various traits on data from the UK Biobank *via* application of snpboost and compared the performance of our approach to the lasso estimates from the BASIL algorithm proposed by Qian et al. as well as to further commonly used methods. On the examined phenotypes we found highly comparable predictive performance while our adapted boosting approach had a tendency to select sparser models compared to the lasso and the other methods. Finally, we illustrate how the framework can be conveniently extended to the classification of binary phenotypes by the incorporation of different loss functions.

## 2 Methods

For n∈N individuals, let $y={\left(y_{1},\ldots ,y_{n}\right)}^{\prime }\in R^{n}$ denote a particular continuous phenotype of interest. Furthermore, let *X*_*j*_ correspond to the genetic variant *j*, for *j* = 1, *…*, *p*. The observed dosage data of *n* individuals is given in the genotype matrix **X** = (*x*_*i*,*j*_) ∈ [0, 2]^*n*×*p*^, where **x**_*j*_ ∈ [0, 2]^*n*^ corresponds to the *j*th column of **X**. We consider a linear regression model

$$E\left(y_{i}|X\right)=\beta_{0}+\overset{p}{\underset{j=1}{\sum }}\beta_{j}x_{i,j},\;i=1,\ldots ,n,$$ (1)

With coefficients $\beta_{0}\in R$ and $\beta ={\left(\beta_{1},\ldots ,\beta_{p}\right)}^{\prime }\in R^{p}$ . The aim is to determine coefficients ${\hat{\beta }}_{0}\in R$ and $\hat{\beta }\in R^{p}$ such that the estimator $\hat{y}={\hat{\beta }}_{0}+X\hat{\beta }$ minimizes the mean squared error of prediction on an independent test set $\text{MSEP}=\frac{1}{n_{\text{test}}}\sum_{i=1}^{n_{\text{test}}}{\left({\hat{y}}_{\text{test},i}-y_{\text{test},i}\right)}^{2}.$ Additionally, one is often interested in relatively sparse models in the sense that only a fraction of the coefficient vector $\hat{\beta }\in R^{p}$ is non-zero.

In high-dimensional settings with *p* > *n* it is not feasible to apply classical estimation techniques like the ordinary least squares estimator. A commonly-used solution is to consider further constraints on the coefficient vector resulting in penalized regression methods including the lasso (Tibshirani, 1996). The lasso incorporates an *L*_1_-penalty on the coefficient vector such that the lasso estimate ${\hat{\beta }}^{\text{lasso}}$ is given by

$${\hat{\beta }}^{\text{lasso}}={\mathrm{argmin}}_{\left(\beta_{0},\beta \right)}\left[\overset{n}{\underset{i=1}{\sum }}{\left(y_{i}-\beta_{0}-\overset{p}{\underset{j=1}{\sum }}\beta_{j}x_{i,j}\right)}^{2}+\lambda \overset{p}{\underset{j=1}{\sum }}|\beta_{j}|\right]$$ (2)

for some *λ* ≥ 0. The explicit *L*_1_-penalization of the coefficient vector leads to shrinkage of the coefficient estimates. In contrast to ridge regression (Hoerl and Kennard, 2000), the use of the *L*_1_-penalty enables to set some parameters exactly to zero corresponding to sparse models. There has been extensive research on the theoretical properties of the lasso including oracle inequalities in high-dimensional settings (e.g., Fu and Knight (2000); Greenshtein and Ritov (2004); Bunea et al. (2007); van de Geer (2008)). Nevertheless, there are situations leading to variable selection problems of the lasso, particularly in the presence of high correlations between signal and noise variables (Hepp et al., 2016). When working with genotype data, high correlations between signal and noise variables might often be present as a result of LD, i.e., genetic variants that have close positions on the DNA strand tend to be highly correlated.

An alternative to explicitly penalized regression methods such as the lasso is statistical gradient boosting (Bühlmann and Hothorn, 2007; Mayr and Hofner, 2018). Gradient boosting requires the specification of a loss function $f\left(y,\hat{y}\right)$ and so-called base-learners *h*_*j*_ that are iteratively fitted to the response. In detail, the aim is again to fit the linear regression model (1) which is performed in an iterative fashion. Starting at iteration *m* = 0 with a starting value ${\hat{y}}^{\left(0\right)}=0$ , the following steps are repeated until a maximum number *m*_stop_ of boosting iterations is reached (Bühlmann and Hothorn, 2007):

1. Set *m*≔*m*+1 and compute the negative gradient vector of the loss function:

$$u^{\left(m\right)}={\left.-\frac{\partial f\left(y,\hat{y}\right)}{\partial \hat{y}}\right|}_{\hat{y}={\hat{y}}^{\left(m-1\right)}}$$

2. Fit every base-learner *h*_*j*_ separately to the negative gradient vector ***u*** ^(*m*)^ and select the best fitting base-learner ${\hat{h}}_{j^{*}}^{\left(m\right)}\left(X_{j}\right)$ .
3. Update the predictor with the learning rate 0 ≤ *ν* ≤ 1: ${\hat{y}}^{\left(m\right)}={\hat{y}}^{\left(m-1\right)}+\nu {\hat{h}}_{j^{*}}^{\left(m\right)}\left(X_{j}\right)$

4. Stop if *m* = *m*_stop_.

**FIGURE 1:**
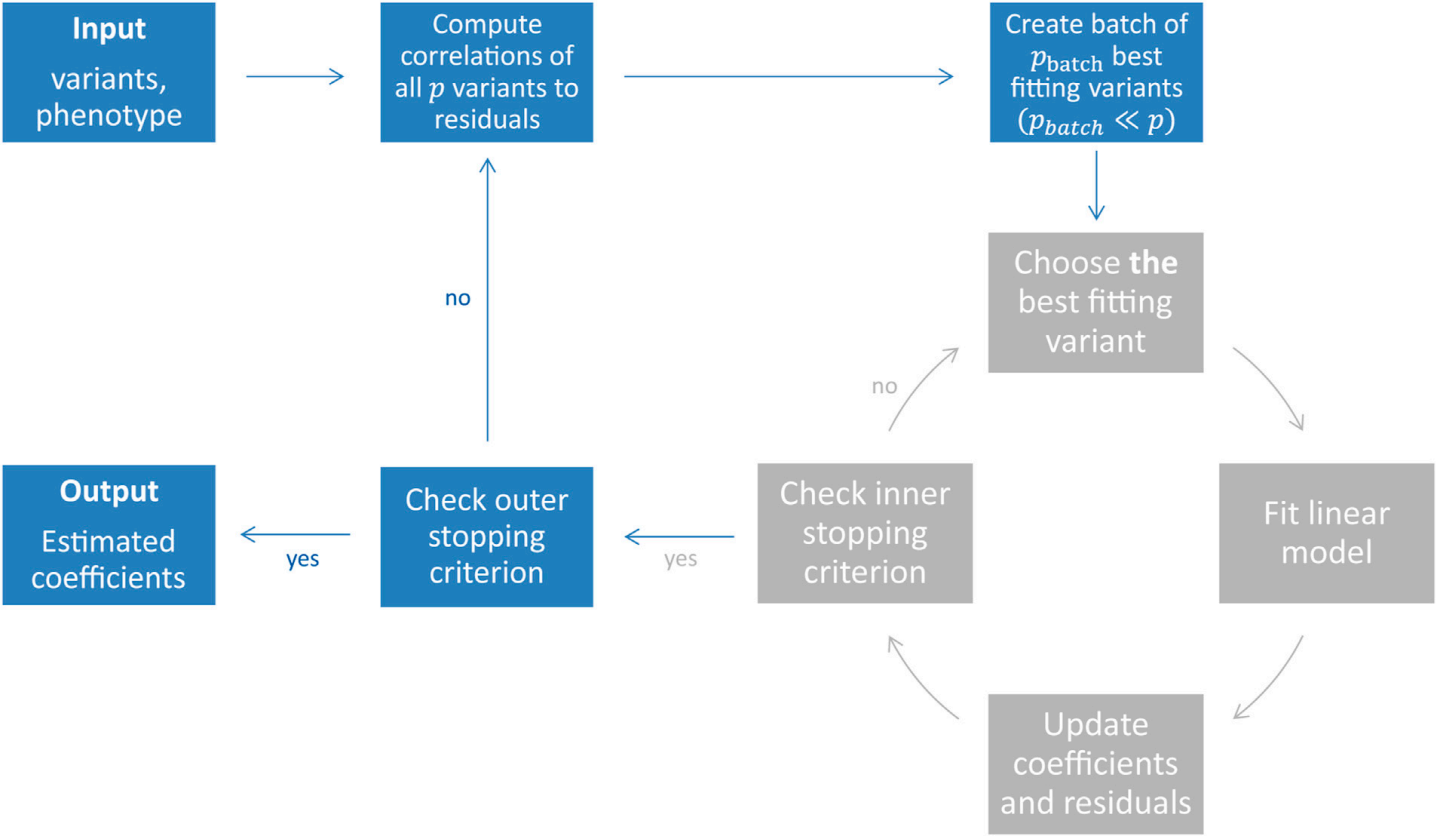
Illustration of the snpboost algorithm. The snpboost algorithm consists of an outer loop to create batches (shown in blue) and an inner loop representing the boosting on one batch (shown in grey).

Stopping the algorithm before it converges (early stopping) leads to implicit regularization and shrinkage of effect estimates. The component-wise *L*_2_-boosting algorithm (Bühlmann and Yu, 2003; Bühlmann and Hothorn, 2007) employs the squared error $f\left(y,\hat{y}\right)=\|y-\hat{y}{\|}_{2}^{2}$ as a loss function (Bühlmann and Yu, 2003) and separate univariate linear regression models of the residuals on the *j*th genetic variant as base-learners (i.e., *h*_*j*_ (*X*_*j*_) = *β*_0_ + *β*_*j*_ *X*_*j*_, for *j* = 1, *…*, *p*). In low-dimensional (*p* < *n*) settings this set-up mimics a classical Gaussian linear model and converges to the least squares solution for large values of *m*_stop_. The general boosting procedure can be interpreted as gradient descent in function space, where the residual vector represents the gradient of the *L*_2_ loss and the function space is provided by the different base-learner solutions (Friedman, 2001; Bühlmann and Yu, 2003; Mayr and Hofner, 2018). The previously described steps transform therefore into the following procedure (shown in grey in Figure 1): The best fitting base-learner in boosting step *m*+1 corresponds to the variant j* with the highest Pearson correlation $\rho \left(x_{j^{*}},r^{\left(m\right)}\right)$ to the residuals $r^{\left(m\right)}=y-{\hat{y}}^{\left(m\right)}$ resulting from the previous boosting step *m*. We then fit a linear regression model of the current residuals ***r*** ^(*m*)^ on the variant j* and update the corresponding coefficient ${\hat{\beta }}_{j^{*}}^{\left(m+1\right)}$ as well as the intercept ${\hat{\beta }}_{0}^{\left(m+1\right)}$ . This is repeated until a maximum number of boosting iterations is reached or any other early stopping criterion is fulfilled. If additional covariates apart from the genetic variants are included in the model, they are treated as mandatory covariates—similar to the intercept. The additional covariates are included in each single base-learner and are hence updated in each boosting step without competing with the genetic variants.

Hepp et al. (2016) investigated the commonalities and differences between the lasso and statistical boosting: while there are (low-dimensional) settings in which the gradient boosting approximates the lasso coefficient paths arbitrarily close when the learning rate *ν* is approaching 0, their results generally differ if the coefficient paths are not monotone. The authors note that, in contrast to the lasso which limits the sum of the absolute values of the coefficients for each penalty parameter *λ* separately, boosting limits the total *L*_1_-arc-length of all coefficient curves (Hepp et al., 2016). Interpreting this as the total absolute distance “travelled” by all coefficients among the coefficient paths through the iterations *m* = 1, …, *m*_stop_, it becomes clear that the solution in a certain iteration depends on all previous solutions of the iterative algorithm. This might lead to more stable pathways particularly in settings with high correlations between independent variables, which is typical for genetic data. Hepp et al. conducted several numerical experiments including high-dimensional settings in which they found similar predictive performance of lasso and boosting. In detail, boosting tended to yield slightly better prediction results while the lasso tended to result in sparser models with faster computations. On the other hand, the boosting algorithm can be easily extended to different response types as well as to different effects, including non-linear and interaction effects. In terms of genetic data, interaction effects can be used to model and identify epistatic effects and gene-environment interactions.

When working on genetic data from large cohort studies we do not only face a high-dimensional setting with *p* > *n* but also a large-scale setting with large sample sizes *n* *and* large numbers of variants *p*. Large-scale settings often lead to extended computational times as well as memory issues. To overcome these and apply statistical boosting on genotype data, we implemented an adapted component-wise *L*_2_-boosting algorithm that is built on the snpnet framework (Qian et al., 2020) and works on batches of variants. To do so, we additionally incorporate a batch-building step before starting the boosting iterations (shown in blue in Figure 1). In this step we extract the *p*_batch_ variants (*p*_batch_ ≪ *p*) with the highest correlation *ρ*(***x***_*j*_, ***r*** ^(*m*)^) to the current residual vector and include them in the batch *B*_*k*_. A maximum number of *m*_batch_ boosting iterations is performed on batch *B*_*k*_ before the next batch is built based on the correlations of all *p* variants to the updated residuals. In total, we fit a maximum of *b*_max_ batches or stop early if an early stopping criterion is fulfilled. The algorithm is summarised in Table 1 and Figure 1.

**TABLE 1:** Definition of the snpboost algorithm without additional covariates. If additional covariates apart from the genetic variants should be included in the model, they are treated as mandatory covariates—similar to the intercept. The additional covariates are included in each single base-learner and are hence updated in each boosting step without competing with the genetic variants.

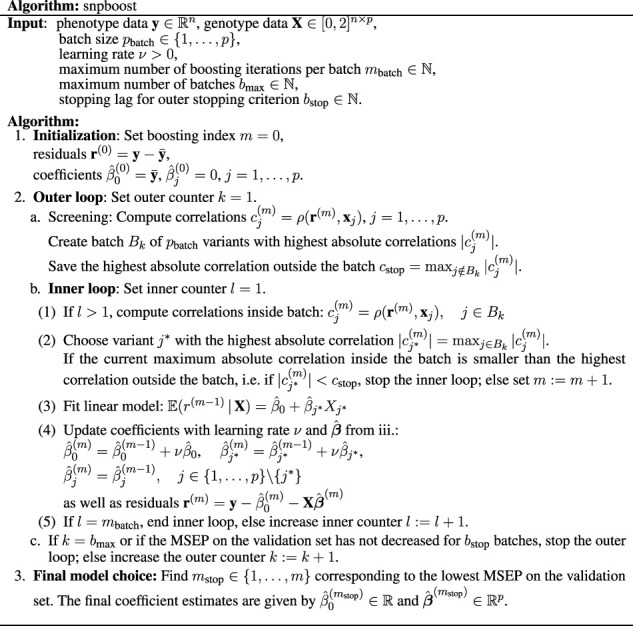

By iteratively working on batches of variants we save computational time and memory because only parts of the variants have to be loaded into memory at once. Additionally, not every step requires the calculation of all potential base-learner solutions and the updated correlations for all variants. By this, we encourage additional sparsity by restricting the search space in terms of the set of available base-learners (as variants not included in the current batch cannot be selected). To examine when a new set of base-learners should be considered, which corresponds to the question when to stop the inner loop (inside the batches) and create a new batch of variants, we incorporated another step: we monitor the correlations of the variants inside a batch to the residuals and compare them to the correlations of variants outside of the batch. When creating a batch *B*_*k*_ we therefore compute and store the highest outer correlation $c_{\text{stop}}≔\max_{j\notin B_{k}}\left|\rho \left(r^{\left(m\right)},x_{j}\right)\right|.$ After each boosting step *m* we check if the greatest absolute correlation of the variants inside the batch *B*_*k*_ to the current residual vector **r** ^(*m*)^ is smaller than *c*_stop_:

$$c_{\text{stop}}>\underset{j\in B_{k}}{\max}\left|\rho \left(r^{\left(m\right)},x_{j}\right)\right|.$$ (3)

If inequality Eq. 3 holds true, we stop the inner loop and create a new batch since a variant outside the batch may provide a better fit to the current residual vector. In the original *L*_2_-boosting without batches, the variant with the highest correlation to the residuals would be chosen in each boosting step. The incorporation of batches in general limits this choice to the variants inside the batch. However, the proposed stopping criterion provides an indication to consider variants outside the batch which may be higher correlated with the current residuals. Actually, if all variants were independent, the proposed stopping criterion would lead to the same choice of variants in each boosting step in snpboost as in the original *L*_2_-boosting. Despite LD, our simulation results show that the proposed stopping criterion yields reasonable variant choices and results in a competitive predictive performance (Section 3.1.1). Additionally, the inner loop is also stopped if the number of updates inside the batch reaches *m*_batch_.

Furthermore, we need to determine after how many batches the algorithm should terminate. In classical statistical boosting the number of boosting iterations is often selected by cross-validation or resampling techniques—mimicking an additional data set to validate the predictive performance of the resulting models. However, if the data set is large enough, one can also directly divide the data into training and validation set. As in Qian et al. (2020), we hence simultaneously monitor the predictive performance of our model on an independent validation set while fitting on the training set. As a validation criterion for the predictive performance we use the MSEP on the validation set. The outer loop consisting of the batch-building step is stopped if the MSEP on the validation set has not decreased for *b*_stop_ batches or after a maximum number of *b*_max_ batches have been processed.

The proposed method is implemented as an add-on to the snpnet package by Qian et al. (2020) in the statistical computing environment R (R Core Team (2021), https://github.com/hklinkhammer/snpboost). While we are also incorporating PLINK 2.0 (Chang et al., 2015) to compute the correlations and build the batches in the outer loop, we replaced the fitting of the lasso by the adapted component-wise *L*_2_-boosting algorithm on the resulting batches (Table 1; Figure 1).

## 3 Empirical results

### 3.1 Simulation study

We conducted a simulation study to investigate the behaviour of the proposed snpboost algorithm in various controlled data scenarios. The simulation study aims at two main goals: first, to examine potential differences in performance compared to the original component-wise *L*_2_-boosting (Bühlmann and Yu, 2003) in smaller settings and, second, to gain insights on how to choose the included hyperparameters in practical situations.

Simulations are based on the UK Biobank genotype data (Bycroft et al., 2018) obtained under application number 81202 combined with simulated phenotypes. We restricted the individuals to white British ancestry and used the PLINK 2.0 function–thin-indiv-count to randomly sample *n* individuals, of which 50%, 20% and 30% were assigned to the training, validation and test set, respectively (Chang et al., 2015; Purcell and Chang, 2015). Then, *p* variants with minor allele frequency not less than 1% were randomly sampled using PLINK 2.0’s–thin-count. Missing genotypes were replaced by the reference allele using the R package bigsnpr (Privé et al., 2018).

Continuous phenotypes were simulated from a linear model with Gaussian distributed noise and effect sizes using bigsnpr. To account for different genetic architectures, we considered varying heritability *h* ^2^ and sparsity *s*, defined as the amount of variance explained by the genetic liability and the proportion of causal variants, respectively. For each setting of *h* ^2^ and *s*, we simulated 100 different datasets. PRS models were derived by snpboost and evaluated by using various metrics regarding the predictive performance and the accuracy of the estimated coefficients. In detail, the predictive performance was measured by the MSEP and the *R* ^2^ value defined as the squared correlation between the predicted and the true phenotype on the independent test set. To assess the computational efficiency we measured the computation time of the algorithm. The accuracy of the resulting estimates was evaluated by the number of included variants in the final model and the mean squared error (MSE) of the estimates as well as the true positive (TP) rates and precision regarding variant selection. Additional results for all considered settings as well as comparisons to snpnet can be found in the Supplementary Material (Supplementary Figures S1–S6).

#### 3.1.1 Comparison to original *L*_2_-boosting in smaller settings

To analyse the performance of snpboost compared to the original component-wise *L*_2_-boosting algorithm (Bühlmann and Yu, 2003), we used a single large batch with batch size *p*_batch_ = *p* in the snpboost algorithm on simulated data with reduced dimensionality. We then compared the results to the ones derived by using smaller batches in terms of predictive performance, computation time, mean squared errors of the estimated coefficients as well as true positive rates and precision regarding variant selection. The simulations were conducted for *n* = 20,000 observations (10,000 training set, 4,000 validation set, 6,000 test set) and *p* = 20,000 variants as well as for varying degrees of heritability and sparsity. To obtain comparable results we chose a fixed number of boosting iterations independent of the batch size *p*_batch_ and a fixed learning rate *ν* = 0.1. For each simulation, 10 CPUs with 1 GB memory each were used.

**FIGURE 2:**
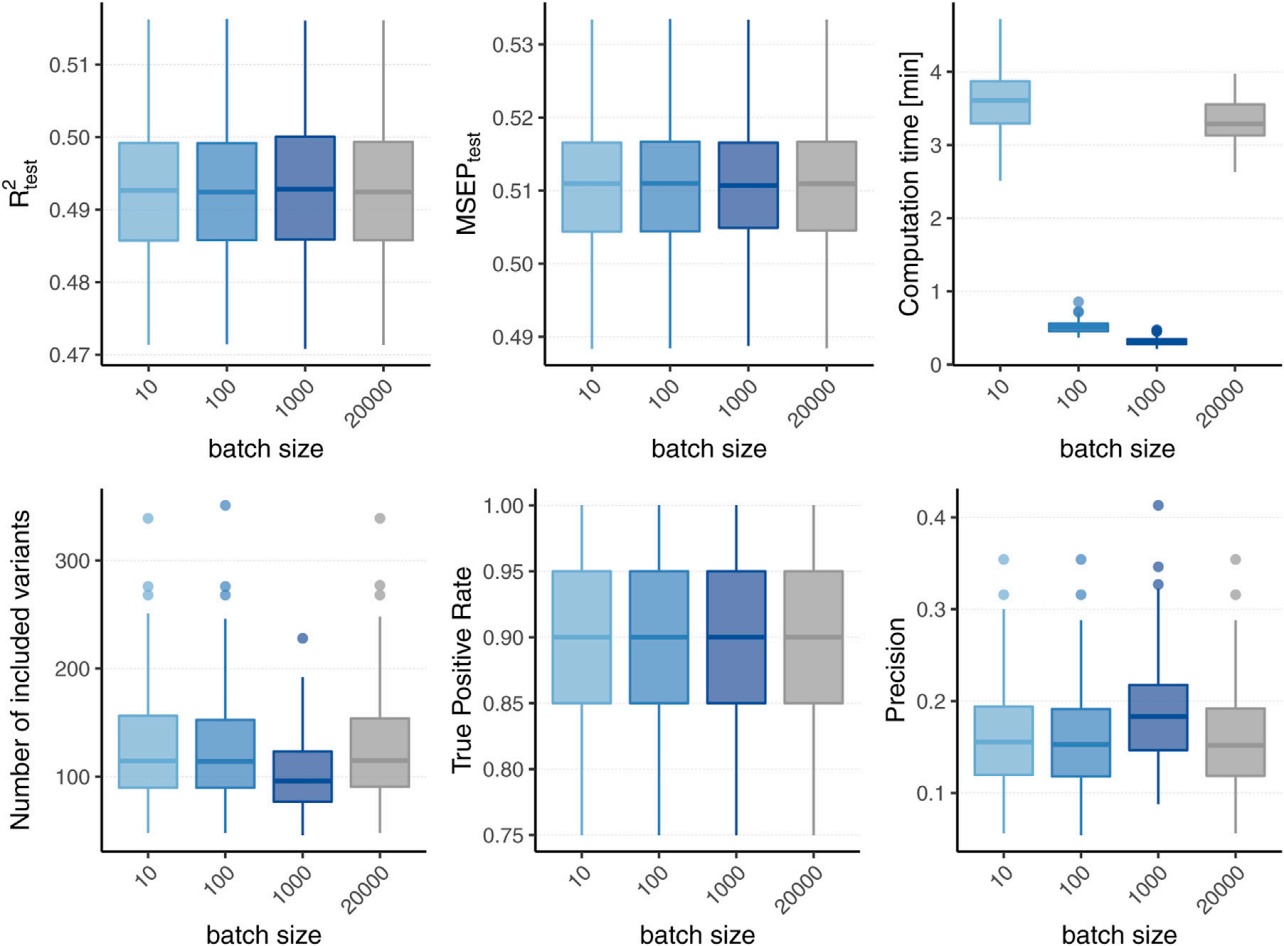
Comparison to original *L*_2_-boosting. Results of 100 simulated phenotypes with heritability *h* ^2^ = 50% and sparsity *s* = 0.1% for *p* = 20,000 variants and *n* = 20,000 individuals (divided into 50% training, 20% validation and 30% test set). Boxplots of the evaluation metrics obtained after 1,500 boosting iterations are shown depending on the batch size. Batch size *p*_batch_ = 20,000 corresponds to the original *L*_2_-boosting (shown in grey).

Figure 2 displays the boxplots of each metric obtained after 1,500 boosting iterations for heritability *h* ^2^ = 50% and sparsity *s* = 0.1%, i.e., 20 influential variants. Incorporating batches did not largely affect the predictive performance in terms of *R* ^2^ and MSEP nor the MSE of the coefficient estimates (MSE results not shown). However, different batch sizes do not always yield the same models as *L*_2_-boosting as can be observed from the number of variants included in the final models. The models resulting from a batch size of *p*_batch_ = 1,000 tend to contain less variants than the ones from the original *L*_2_-boosting (batch size *p*_batch_ = 20,000). This could be explained by the reduced search space in each boosting step and a trade-off between exploration (genome-wide search) and exploitation (search inside the batch). As a consequence, variants within the batch that are already in the model are more often updated instead of including new variants outside of the batch. The same holds true when comparing the number of chosen variants for batch size *p*_batch_ = 1,000 to smaller batch sizes (i.e., *p*_batch_ = 10 and *p*_batch_ = 100). As all models tend to overestimate the number of influential variants, the lower number of selected variants for batch size *p*_batch_ = 1,000 corresponds to a higher precision since less false positives are included. The fact that the other metrics remain almost constant suggests that either only variants with very small effects are not included when using a larger batch size or the variants that are updated are highly correlated with the ones not included. Furthermore, incorporating batches in the algorithm has a major effect on the computation time. To interpret the results shown in Figure 2 it is important to understand the two drivers of the computation time. On the one hand, it increases with the number of correlations that have to be calculated in each boosting step which explains the increased computation time of the original *L*_2_-boosting (i.e., a batch size of *p*_batch_ = 20,000 and 20,000 computed correlations in each boosting step) compared to smaller batch sizes such as *p*_batch_ = 100 and *p*_batch_ = 1,000. On the other hand, reading the genotype data from disk when building the batches also increases the computation time leading to a higher computation time for smaller batches with *p*_batch_ = 10 for which more reads-from-disk have to be carried out. The varying computation times therefore reflect a trade-off between the number of correlations computed in each boosting step and the number of created batches.

In summary, the incorporation of batches in the boosting algorithm did not affect the predictive performance of the model in our scenarios, while computation time was substantially reduced. However, snpboost does not always lead to the same models as the original *L*_2_-boosting algorithm, in particular in terms of the included variants and sparsity. The results for further settings with different heritability and sparsity were comparable and can be found in the Supplementary Material.

#### 3.1.2 Choice of hyperparameters for large-scale applications

The proposed snpboost algorithm includes various hyperparameters, namely the batch size *p*_batch_, the learning rate *ν*, the maximum number of boosting iterations per batch *m*_batch_, the maximum number of processed batches *b*_max_ and the stopping lag for the outer early stopping criterion *b*_stop_. In this section we discuss default values for the hyperparameters to facilitate the applicability of the algorithm in practice. The majority of these parameters do not need to be tuned but can be specified with reasonable default values, e.g., based on results from the literature and experience with the original boosting algorithm. For the remaining ones (*p*_batch_ and *b*_stop_) we examine how they influence the computational and predictive performance of snpboost in a simulation study.

The choice of the learning rate *ν* can be leaned on widely-used boosting algorithms. A rather small learning rate prevents boosting algorithms from overfitting on single base-learners and is therefore favorable regarding predictive performance. Nevertheless, a smaller learning rate will increase the number of needed boosting iterations to fit the full effect of the base-learners and simultaneously increase the algorithm’s computation time. Widely used R packages such as mboost (Bühlmann and Hothorn, 2007; Hothorn et al., 2010) and xgboost (Chen and Guestrin, 2016) use default learning rates of 0.1 and 0.3, respectively. As the effect of the learning rate will be comparable in the proposed adapted boosting algorithm, we decided to specify a fixed default value of *ν* = 0.1 in all our simulations. For the batch-related hyperparameters we varied the batch size *p*_batch_ over a range of possible values namely *p*_batch_ ∈ {10, 100, 1,000, 5,000} to analyse its effect. For each batch we allow a maximum number of boosting iterations *m*_batch_ equivalent to the batch size *p*_batch_. Since we specified the learning rate with a rather small fixed value and due to the correlation-based early stopping criterion, this choice should prevent the algorithm from overfitting on one batch. If one or more variants inside the batch are still among the most influential ones out of all variants they will also be included in the next batch. For the outer stopping criterion we specified a large maximum number of batches *b*_max_ = 20,000 to ensure that the algorithm terminates even in case the MSEP on the validation set has not decreased for *b*_stop_ batches. Since we do not want the algorithm to stop too early and simultaneously minimize the computation time, in our simulations we consider the choices *b*_stop_ = 2 and *b*_stop_ = 10. We then fitted PRS models using snpboost with the previously described hyperparameters. For the computations we used 10 CPUs with 2 GB RAM each.

**FIGURE 3:**
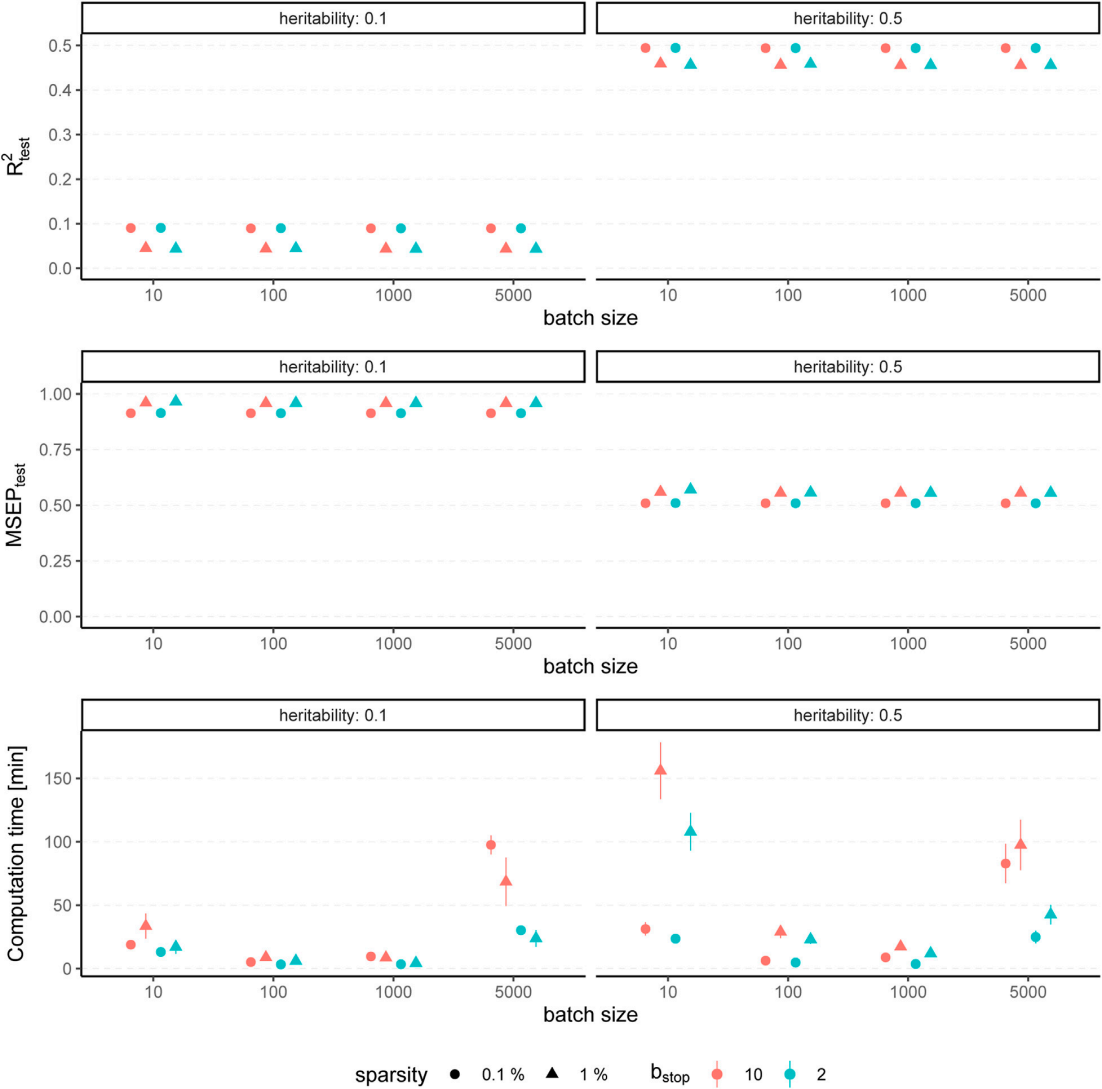
Predictive performance for varying batch size and stopping criteria. Results of 100 simulated phenotypes with heritability *h* ^2^ ∈ {10%, 50%}, sparsity *s* ∈ {0.1%, 1%} and *b*_stop_ ∈ {2, 10} for *p* = 100, 000 variants and *n* = 100, 000 individuals (divided into 50% training, 20% validation and 30% test set). Mean and standard deviation of the evaluation metrics are shown depending on the batch size.

**FIGURE 4:**
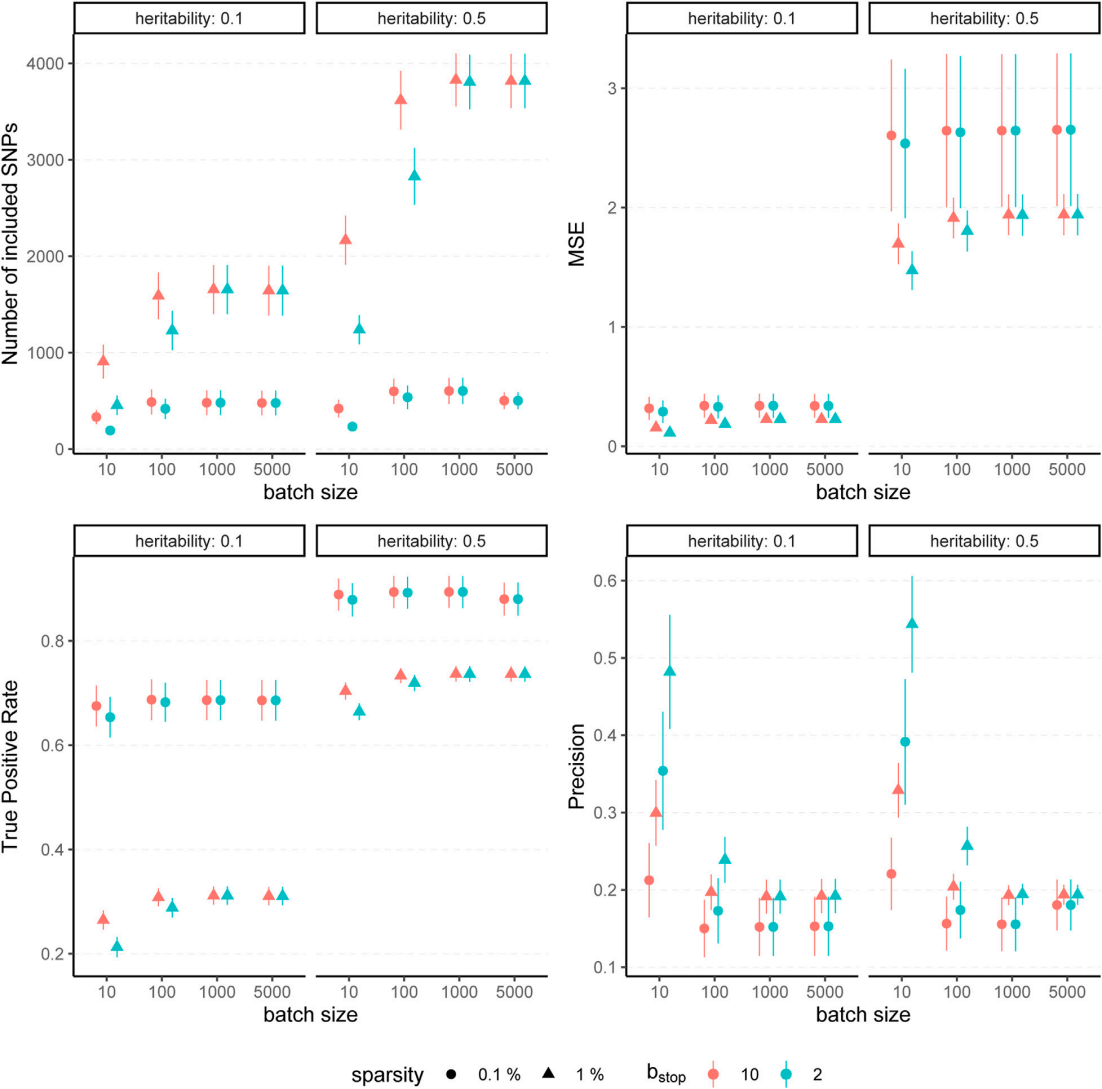
Evaluation metrics of the estimated coefficients for varying batch size and stopping criteria. Results of 100 simulated phenotypes with heritability *h* ^2^ ∈ {10%, 50%}, sparsity *s* ∈ {0.1%, 1%} and *b*_stop_ ∈ {2, 10} for *p* = 100,000 variants and *n* = 100,000 individuals (divided into 50% training, 20% validation and 30% test set). Mean and standard deviation of the evaluation metrics are shown depending on the batch size.

The results for simulated phenotypes with 10% and 50% heritability are shown in Figure 3 and Figure 4. Results for further degrees of heritability can be found in the supplement. Independently of the heritability and the sparsity of the simulated data, the predictive performance was not affected in our settings by varying batch sizes in terms of *R* ^2^ and MSEP. However, the computation time differed crucially, resulting in considerably higher values for rather small (*p*_batch_ = 10) or rather large (*p*_batch_ = 5,000) batches. Furthermore, larger batches led to a higher number of included variants in the final model. This effect was stronger for phenotypes which have a less sparse genetic architecture and associated with a later stopping of the algorithm, i.e., more boosting steps were required to derive the final model. A higher number of variants in the final model was associated with a slightly higher MSE of the coefficients as well as higher true positive rates on the one hand but also smaller precision on the other hand. As expected, a higher *m*_stop_ increased the computation time of the fitting process for all batch sizes. In contrast, there was no considerable effect on the predictive performance. However, *b*_stop_ = 2 and *b*_stop_ = 10 had an impact on the coefficient estimates as can be seen in Figure 4, e.g., by a tendency to include more variants in the model when choosing *b*_stop_ = 10. This tendency was only apparent for batch sizes *p*_batch_ < 1,000, suggesting that for larger batches the choice of *b*_stop_ is only of minor importance for both, prediction performance and coefficient estimates. The results clearly indicate that a more favorable signal-to-noise ratio (i.e., a higher heritability) and less influential variants (i.e., a higher sparsity) are in general beneficial for the performance of our approach. For phenotypes with a sparser genetic architecture, the considered evaluation metrics tended to show less variability.

In summary, the choice of the hyperparameters had no major influence on the predictive performance measures *R* ^2^ and MSEP but on the computation time, which was lowest for medium size batches (100 ≤ *p*_batch_ ≤ 1,000). The accuracy of the coefficient estimates measured *via* MSE, TP and TN rate varied with the batch size, as larger batches tended to lead to more (true positive) variants included in the final model, but also to a slightly higher MSE and a smaller TN rate. While the differences in MSE, TP, and TN rate were only small, smaller batches yielded sparser models in particular for phenotypes with a high heritability.

To conclude, batch sizes of 100 ≤ *p*_batch_ ≤ 1,000 seem to be the most favorable regarding the computation time and the other evaluation metrics. We propose a batch size of *p*_batch_ = 1,000 as the default value because the results suggest less dependency on the *b*_stop_ parameter than for a batch size of 100 variants. Accordingly, we recommend a default value of *b*_stop_ = 2 to keep the computation time as low as possible. In practice, genotype data most often contain more than 100,000 variants, which further supports the choice of *p*_batch_ = 1,000 with regard to the computation time. Although our simulation study suggests that those default values should provide reasonable results in most cases, it is recommendable to take the genetic architecture of the examined phenotype as well as the main aim of the analysis into account. Phenotypes with a high expected heritability might be better fitted by using smaller batches, while for phenotypes with many causal variants larger batches might be favorable to increase the TP rate. If one is interested in extremely sparse models identifying only the most-informative variants one could also try to use smaller batches to avoid an overestimation of the number of causal variants.

### 3.2 Application to the UK Biobank

We applied our proposed method on data from the UK Biobank resource under Application Number 81202. Besides the validation of the results from the previous section, we compared our boosting models fitted *via* the proposed snpboost approach to the ones derived by fitting the lasso *via* the BASIL algorithm implemented in the snpnet package, which have already been shown to outperform commonly-used PRS models for various traits (Qian et al., 2020). Furthermore, we compared our results to PRScs (Ge et al., 2019), LDpred2 (Privé et al., 2021) and SBayesR (Lloyd-Jones et al., 2019), which are based on summary statistics, as well as to multivariable methods *via* LDAK (Zhang et al., 2021) based on Bolt-LMM (Loh et al., 2015), Ridge Regression (Henderson, 1950) and BayesR (Moser et al., 2015).The UK Biobank (UKBB, Bycroft et al., 2018) is a large-scale prospective cohort study including more than half a million participants from the United Kingdom aged between 40 and 69 years when recruited. The database comprises genome-wide genotype data of each individual as well as various in-depth phenotypic information such as biological measurements as well as blood and urine biomarkers. The data have been collected since 2006 and are continually updated with follow-up data.

Our aim is to estimate PRS for various phenotypes, covering several heritability and sparsity levels. The heritability of a trait is an upper bound for the predictive performance based on genotype information. Thus, we used the analyses of Tanigawa et al. (2022) as a proxy and specifically considered five appropriate continuous phenotypes: standing height in cm (UKBB field 50), LDL-cholesterol in mmol/l (UKBB field 30780), blood glucose level in mmol/L (UKBB field 30740), lipoprotein A in nmol/L (UKBB field 30790) and BMI in kg/m^2^ (UKBB field 21001).

Height and BMI are quantitative traits with a relatively high heritability and a rather polygenic structure. Twin-studies estimated a heritability of approximately 69% for height and 42% for BMI after adjusting for covariates (Hemani et al., 2013). For a long time, genetic models could not explain this estimated heritability, a phenomenon known as “missing heritability” (Maher, 2008; Gibson, 2010). More recent studies have indicated that this may be primarily due to many influential common variants with small effect sizes (Yang et al., 2010; Wood et al., 2014; Yang et al., 2015) underlining the high polygenicity of those traits. In contrast to this, the distribution of the biomarker lipoprotein A, which is a strong risk factor for coronary heart disease, is mainly explained by variants in the LPA gene on chromosome 6 (Kronenberg and Utermann, 2013). Thus, we expect a sparse PRS with a relatively high prediction accuracy for this trait. For LDL-cholesterol it is known that it is associated with several genes such as LDLR and PCSK9 (Sanna et al., 2011; Sabatine, 2019). Therefore, we expect signal in several genomic regions. Recent studies compared different approaches including the lasso to derive PRS, and found that multivariable methods can reach a predictive performance of up to 20% (Maj et al., 2022; Tanigawa et al., 2022). As in previous works (Sinnott-Armstrong et al., 2021), we adjusted the measured LDL-cholesterol value by a factor of 0.684 for individuals taking statins lowering the blood lipid. For blood glucose we are not aware of a genetic impact and also Tanigawa et al. (2022) found the genetic background only explaining a small fraction (less than 2%) of the biomarker’s variance.

Out of the over 500,000 individuals from UK Biobank we filtered for unrelated (based on UKBB resource 668) individuals with self-reported white British ancestry (UKBB field 21000) and available data for all chosen phenotypes, resulting in *n* = 262,171 observations. Additionally, the covariates age and sex as well as the first ten principal components of the genotype matrix are available. We randomly divided the data set into training (*n*_train_ = 157,204), validation (*n*_val_ = 52,416) and test set (*n*_test_ = 52,551). We used genome-wide genotype data and filtered for variants with a genotyped rate of at least 90% and a minor allele frequency of at least 0.1%, resulting in *p* = 562,723 genetic variants. Missing genotypes are imputed by the corresponding mean of the complete observations.

For both the boosting and lasso approaches, we first estimated a PRS using only the genotyped variants as predictors. We used the training set to fit the model and the validation set to simultaneously monitor the predictive performance for choosing the main tuning parameters of the algorithms (i.e., the number of iterations for boosting and the penalty parameter for the lasso). To fit the lasso we used the R package snpnet (Qian et al., 2020) with the provided default hyperparameters. Following the results of our simulation study, for the snpboost algorithm we chose a batch size of *p*_batch_ = 1,000 variants, a learning rate of *ν* = 0.1 and an outer stopping lag of *b*_stop_ = 2 batches. Using the resulting estimated $\hat{\text{PRS}}$ we fitted two linear models on the combined training and validation set, namely the first one (*M*_PRS_) incorporating only the PRS as a single predictor variable:

$$M_{\text{PRS}}:Y=\gamma_{0}+\gamma_{\text{PRS}}\hat{\text{PRS}}$$ (4)

and the second one (*M*_*f*_) including the first ten principal components, sex and age as additional covariates:

$$M_{f}:Y=\gamma_{0}+\gamma_{\text{PRS}}\hat{\text{PRS}}+\gamma_{1}{\text{PC}}_{1}+\cdots +\gamma_{10}{\text{PC}}_{10}+\gamma_{\text{sex}}\text{sex}+\gamma_{\text{age}}\text{age}.$$ (5)

To measure the actual benefit in accuracy of including a PRS in the prediction model, we also fitted a model including only covariates (*M*_*c*_):

$$M_{c}:Y=\gamma_{0}+\gamma_{1}{\text{PC}}_{1}+\cdots +\gamma_{10}{\text{PC}}_{10}+\gamma_{\text{sex}}\text{sex}+\gamma_{\text{age}}\text{age}.$$ (6)

Finally, we also included the covariates in the fitting process to derive the PRS, corresponding to the model *M*_*PRS,c*_:

$$M_{\text{PRS},c}:Y=\beta_{0}+\beta_{\text{PC}1}{\text{PC}}_{1}+\cdots +\beta_{\text{PC}10}{\text{PC}}_{10}+\beta_{\text{sex}}\text{sex}+\beta_{\text{age}}\text{age}+\overset{p}{\underset{j=1}{\sum }}\beta_{j}X_{j}.$$ (7)

All models were evaluated on the independent test set and compared with respect to their predictive performance, computational efficiency and sparsity. To measure the predictive performance we used the *R* ^2^ value on the test set given by the squared correlation between the observed and predicted phenotypes as well as the root mean squared error of prediction (RMSEP). The computational efficiency was measured as the computation time in minutes of the respective algorithm and the sparsity is given by the number of included variants in the final PRS. All computations were conducted on a computer cluster with 16 CPUs and 2 GB RAM each. The derivation of the PRS by the use of further methods (namely PRScs, LDpred2, SBayesR, Bolt-LMM, Ridge Regression and BayesR) was based on the same training and validation data and is described in the Supplementary Material. All models were tested on the same independent test set.

**FIGURE 5:**
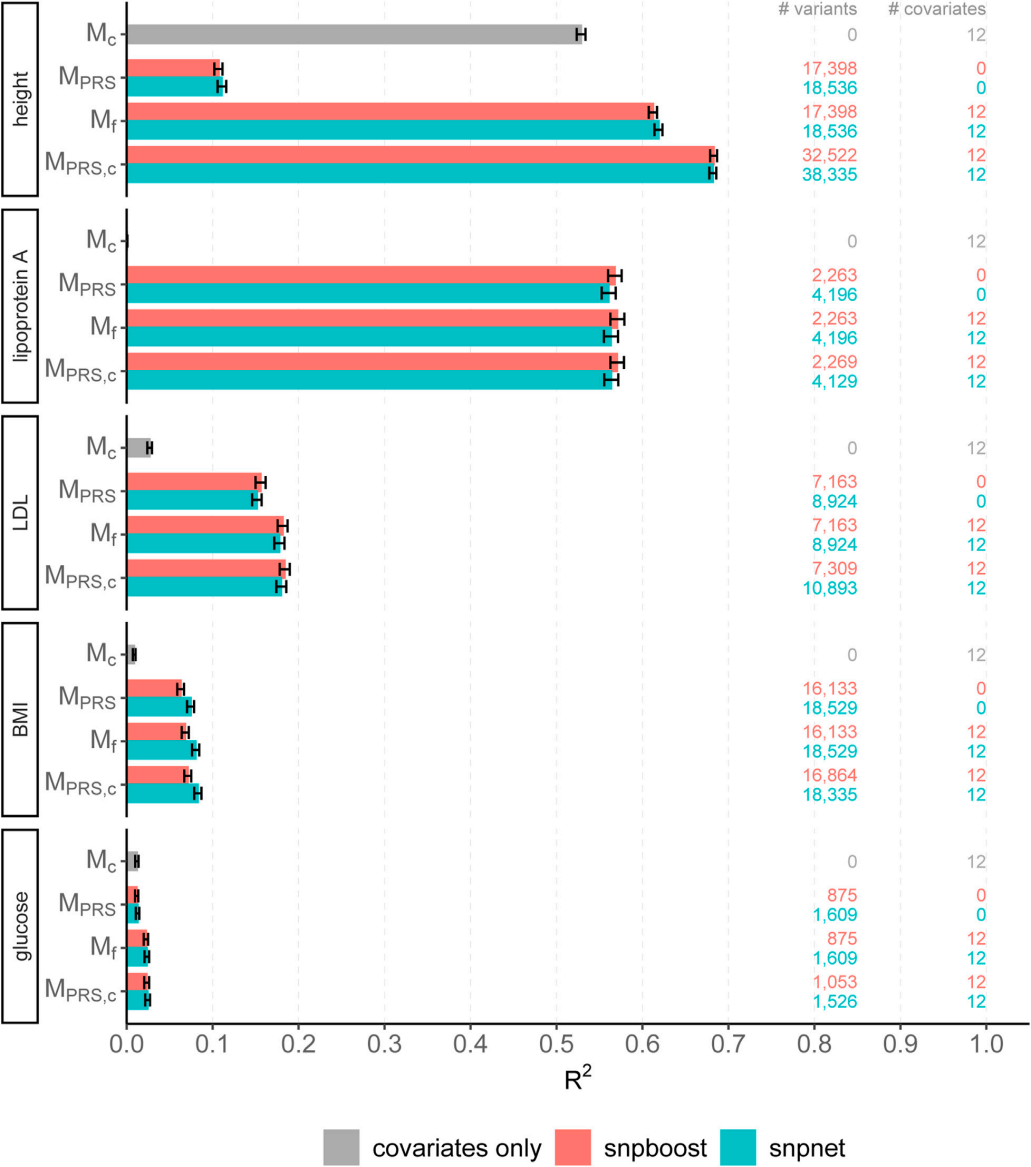
Comparison of predictive performance of snpnet and snpboost for five continuous phenotypes from the UKBB. Results of the covariate-only model (*M*_*c*_; grey bars) and multivariable polygenic models with and without inclusion of the covariates derived by lasso (snpnet; petrol-colored bars) and statistical boosting (snpboost; red bars) for the prediction of five phenotypes from the UKBB. The barplots show the predictive performance (*R* ^2^) on the test set of 52,551 unrelated white British individuals. *M*_PRS_ corresponds to a linear model incorporating the PRS as a single predictor variable and *M*_*f*_ to a linear model incorporation sex, age and the first ten principal components as additional covariates. *M*_PRS,*c*_ includes the covariates already in the fitting process of the PRS. Bootstrapped 95% confidence intervals are indicated by error bars. Furthermore, information on the number of selected genetic variants (# variants) and the number of additionally included covariates (# covariates) is given.

The results of snpboost as well as of snpnet for all phenotypes are given in Figure 5 and Table 2. The resulting RMSEP is shown in Supplementary Figure S7. Overall, snpnet and snpboost yield comparable results regarding the predictive performance, without one approach being consistently superior to the other. Both the resulting *R* ^2^ and RMSEP are very close. Furthermore, the shown *R* ^2^ values are in line with previously reported *R* ^2^ resulting from snpnet, which has been shown to be highly competitive to various other (univariate) PRS methods (Qian et al., 2020; Li et al., 2022; Tanigawa et al., 2022). The PRS estimated *via* snpnet and snpboost both clearly increase the predictive performance compared to the covariates-only model *M*_*c*_ for all shown phenotypes. With respect to sparsity, our boosting approach tends to select less variants (on average 26% less variants compared to the lasso). The computation time of both approaches is highly dependent on the genetic architecture, i.e., the heritability and sparsity of the phenotype. In particular, a higher and more polygenic signal tends to lead to longer computation times. In case of fitting the PRS based solely on the genotype data and including the covariates in a subsequent linear model, snpboost tends to be faster than snpnet; however, the computation times for snpboost increase substantially when including covariates in the fitting process for LDL-cholesterol and height. This is partly due to more coefficients being fitted and updated in each boosting step and partly due to larger PRS models resulting from more boosting steps. Nevertheless, the models are still fitted in reasonable time using our batch-based approach. As described in Hepp et al. (2016), boosting is generally expected to be slower than the lasso, which can only be observed for less sparse models in the examined phenotypes. In general, the model *M*_PRS,*c*_ outperforms the model *M*_*f*_ regarding the predictive performance, implying that including the covariates already in the fitting of the PRS is favorable regarding the detection of the genetic signal. However, the effect is only substantial for phenotypes with a high association to covariates (i.e., height). Furthermore, the model *M*_PRS,*c*_ tends to select more variables than estimating the PRS based only on the genotypes (*M*_PRS_) and the computation time is considerably increased when using the snpboost approach. Therefore, it might be advisable to only consider the covariates in the fitting process if there is a large association already in the covariates-only model.

**TABLE 2:** Comparison of computational efficiency of snpnet and snpboost on eight phenotypes from the UKBB. Computational times of the algorithms snpnet and snpboost for multivariable polygenic models with and without inclusion of the covariates for the prediction of eight phenotypes from the UKBB. *M*_PRS_ corresponds to the application of the algorithms without including covariates and *M*_PRS,*c*_ to the inclusion of the covariates sex, age and the first ten principal components. The experiments were run on 16 CPUs with 2 GB RAM each.

		Computation time in minutes	
Phenotype	Model	snpnet	snpboost
Height	*M*_PRS_	132.44	116.65
Height	*M*_PRS,*c*_	97.49	299.98
BMI	*M*_PRS_	54.36	94.34
BMI	*M*_PRS,*c*_	54.81	156.49
LDL	*M*_PRS_	37.92	50.64
LDL	*M*_PRS,*c*_	45.61	64.27
glucose	*M*_PRS_	14.86	11.38
glucose	*M*_PRS,*c*_	14.71	16.33
lipoprotein A	*M*_PRS_	28.99	25.08
lipoprotein A	*M*_PRS,*c*_	33.67	30.14
asthma	*M*_PRS_	3.97	5.31
asthma	*M*_PRS,*c*_	4.21	6.63
coeliac	*M*_PRS_	3.11	5.46
coeliac	*M*_PRS,*c*_	5.00	6.69
HBP	*M*_PRS_	46.63	90.27
HBP	*M*_PRS,*c*_	30.07	181.23

**FIGURE 6:**
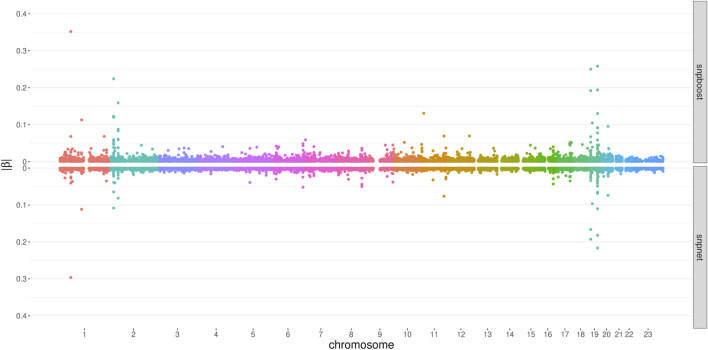
Absolute values of coefficient estimates for PRS models for LDL-cholesterol derived by boosting (snpboost) and lasso (snpnet) in dependence of the genomic position of the variants.

Figure 6 displays the absolute values of the resulting estimated non-zero coefficients for LDL-cholesterol for the boosting and lasso approaches. Both tend to detect variants with higher effect sizes in the same genetic regions, e.g., at chromosome 2 and chromosome 19. In total, there are 3,030 genetic variants that are present in both PRS, out of 7,163 variants selected by snpboost and 8,924 variants selected by snpnet. While snpboost results in less variants, the included variants have larger effect sizes and less variants with very small effect sizes close to zero are included in the model. Supplementary Figure S8 displays the coefficients again with shared variants marked in black. All SNPs with comparably high effect sizes in the snpnet PRS are included in both models but the snpboost PRS incorporates further SNPs with stronger effects. The results are similar for the other phenotypes and included in the Supplementary Material. In conclusion, the snpboost PRS tends to include less variants in total, but more variants with comparably high effect sizes corresponding to less shrinkage for the variants included in the model compared to the lasso.

The Supplementary Material comprises results for comparisons to further commonly used methods to derive PRS (Supplementary Tables S1, S2). Our proposed algorithm yielded consistently higher prediction performance compared to the summary statistics based PRScs and LDpred2 methods; furthermore, it yielded competitive results compared to summary statistics based SBayesR and four different multivariable approaches, while tending to select the sparsest models.

## 4 Extension to binary data

While traits like blood biomarkers or physical measurements are often quantitative, it is, for example, also of interest to predict the probability of the occurrence of a disease for a particular patient. In this case we deal with binary data *y*_*i*_ ∈ {0, 1} and proceed as in a logistic regression by modelling the logit of the expected value as a linear model

$$\text{logit}\left(\text{P}\left(y_{i}=1|X\right)\right)=\ln\left(\frac{\text{P}\left(y_{i}=1|X\right)}{1-\text{P}\left(y_{i}=1|X\right)}\right)=\beta_{0}+\overset{p}{\underset{j=1}{\sum }}\beta_{j}x_{i,j},\;i=1,\ldots ,n.$$ (8)

The estimated probability $\hat{p_{i}}\left(X\right)=\text{P}\left(y_{i}=1|X\right)$ is then given by

$$\hat{p_{i}}\left(X\right)=\text{P}\left(y_{i}=1|X\right)=\frac{\exp\left({\hat{\beta }}_{0}+\sum_{j=1}^{p}{\hat{\beta }}_{j}x_{i,j}\right)}{1+\exp\left({\hat{\beta }}_{0}+\sum_{j=1}^{p}{\hat{\beta }}_{j}x_{i,j}\right)}.$$ (9)

To fit binary outcomes *via* boosting we replace the *L*_2_ loss by the log loss

$$f_{\ln}\left(y,\hat{p}\right)=-\frac{1}{n}\overset{n}{\underset{i=1}{\sum }}y_{i}\ln\left({\hat{p}}_{i}\right)+\left(1-y_{i}\right)\ln\left(1-{\hat{p}}_{i}\right).$$ (10)

Note that following this new loss function, the gradient is no longer represented by the residuals. The base-learners are hence fitted now to the first derivative of the loss function in Eq. 10. Consequently, batches are built out of the *p*_*b*_ *atch* variants with the highest absolute correlation to the first derivative of the loss in Eq. 10 instead of the residual. However, the other components of the algorithm including the base learners remain unchanged. We also keep the hyperparameters derived in Section 3.1.2 fixed. We applied the extended algorithm on data of the UKBB for three binary phenotypes: the occurrence of asthma (UKBB field 22127), coeliac disease (UKBB field 21068) and high blood pressure (UKBB field 6150). All three traits are associated to many environmental factors but also have a genetic component (Arora and Newton-Cheh, 2010; Trynka et al., 2010; Yang et al., 2017; El-Husseini et al., 2020). Tanigawa et al. (2022) estimated high blood pressure to be a rather polygenic trait while the genetic component of asthma and coeliac disease is determined by fewer common variants.

We incorporated unrelated individuals of white British ancestry in our analysis and divided the samples randomly into training, validation and test sets. In total we used 8,397 cases (*n*_train_ = 4,266, *n*_val_ = 1,709 and *n*_test_ = 2,522) and 58,428 controls (*n*_train_ = 29,079, *n*_val_ = 11,707, *n*_test_ = 17,642) for asthma, 1,793 cases (*n*_train_ = 882, *n*_val_ = 361 and *n*_test_ = 550) and 92,646 controls (*n*_train_ = 46,234, *n*_val_ = 18,449, *n*_test_ = 27,963) for coeliac disease and 71,235 cases (*n*_train_ = 35,720, *n*_val_ = 14,210 and *n*_test_ = 21,305) and 190,422 controls (*n*_train_ = 94,740, *n*_val_ = 38,246, *n*_test_ = 57,436) for high blood pressure.

The applicability to binary traits was also one of the first extension of snpnet and Qian et al. (2020) showed impressive results for a number of binary traits. Due to that, we again also apply snpnet to the same data to evaluate the quality of our results.

**FIGURE 7:**
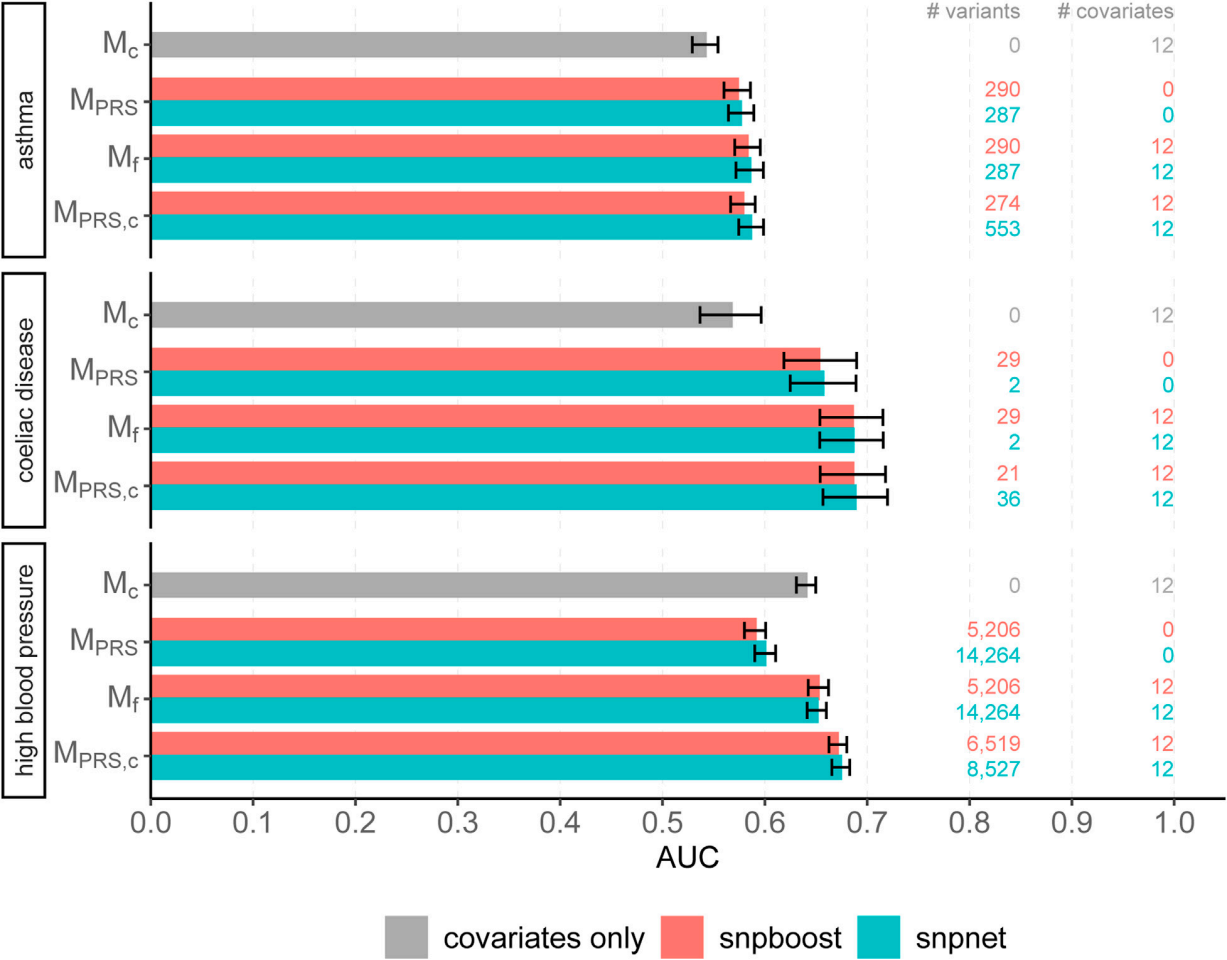
Comparison of predictive performance of snpnet and snpboost for three binary phenotypes from the UKBB. Results of the covariate-only model (*M*_*c*_; grey bars) and multivariable polygenic models with and without inclusion of the covariates derived by lasso (snpnet; petrol-coloured bars) and statistical boosting (snpboost; red bars) for the prediction of three binary phenotypes from the UKBB. The barplots show the AUC on the test set of 20,164 (asthma), 28,513 (coeliac disease) and 78,741 (high blood pressure) unrelated white British individuals. *M*_PRS_ corresponds to a logistic regression model incorporating the PRS as a single predictor variable and *M*_*f*_ to a logistic regression model incorporating sex, age and the first ten principal components as additional covariates. *M*_PRS,*c*_ includes the covariates already in the fitting process of the PRS. Bootstrapped 95% confidence intervals are indicated by error bars. Furthermore, information on the number of selected genetic variants (# variants) and the number of additionally included covariates (# covariates) is given.

We evaluated the accuracy of the resulting predictions on the test set using both the log loss as well as the AUC. Results are shown in Figure 7 and in Supplementary Figure S17. The overall predictive performance is comparable for all three phenotypes. For high blood pressure with a polygenic genetic component snpboost yields a sparse model with a high predictive performance. For sparse binary phenotypes as asthma and coeliac disease, snpboost and snpnet yield similar sparse models. The result for coeliac disease, which appears to be rather oliogenic than polygenic, for snpnet is outstanding, but in line with the results of Tanigawa et al. (2022). Nevertheless, also snpboost also estimates a very sparse PRS with a high predictive performance. Table 2 illustrates the computation time for binary data of snpnet and snpboost on a computer cluster with 16 CPUs and 2 GB RAM each. Both, snpboost and snpnet yield very limited computation times, with snpnet being faster.

In summary, this illustrates how easily and conveniently the snpboost framework can be extended to different data types by incorporating different loss functions. Even though we simply plugged in the log loss and did not optimize the hyperparameters such as the batch size or the learning rate of our algorithm for binary data, snpboost yields a competitive predictive performance compared to the BASIL algorithm.

## 5 Discussion

In this work we have proposed a new methodological framework to derive multivariable PRS models *via* applying a statistical boosting approach directly on genotype data. Currently, PRS are most often built based on summary statistics from GWAS that were estimated by simple and univariate linear regression models (Choi et al., 2020). This methodologically simple approach is mainly justified by the computational hurdle resulting from the ultra-high-dimensionality of the genotype data. For example, in the past it had been unfeasible to fit a lasso model on the complete genotype data due to the high computational complexity. To overcome this, Mak et al. (2017) developed lassosum, an approach to approximate the lasso path by only using summary statistics and LD reference panels. However, recently published works provided methods to enable statistical modelling by penalized multivariable regression approaches on genotype data (Privé et al., 2018; Qian et al., 2020). Qian et al. demonstrated that lasso-based PRS were able to outperform several PRS derived by methods based on univariate summary statistics (Qian et al., 2020). First approaches to apply statistical boosting on genotype data employed a three-step-approach to fit multivariable PRS (Maj et al., 2022): first, variants are pre-filtered based on their univariate associations with the examined phenotype. Second, statistical modelling and variable selection approaches such as AdaSub (Staerk et al., 2021) and boosting with probing (Thomas et al., 2017) are used to identify the informative variants on blocks of variants in LD. Finally, a multivariable PRS based on the selected variants is constructed *via* component-wise *L*_2_-boosting (Bühlmann and Hothorn, 2007). While this approach yielded particularly sparse models and could compete with common methods like clumping and thresholding (Euesden et al., 2014), lasso *via* snpnet yielded more accurate results regarding the predictive performance which is usually the main objective of PRS modelling.

In the present article we introduced the boosting algorithm snpboost that works on smaller batches of variants similar to the BASIL algorithm. Our framework enables the application of statistical boosting directly on the complete original genotype data. In a smaller but still high-dimensional simulation setting, we were able to show that the adapted boosting algorithm yields similar performance to the original component-wise *L*_2_-boosting, indicating that we do not lose predictive performance due to the incorporation of batches. In a further setting with more realistic dimensionality we have derived appropriate default values for the application of snpboost on large-scale data. We were able to show that the specified default values resulted in models with good performance in most cases but also gave advice on how to adapt them based on the genetic architecture of the examined phenotype and the specific research questions.

We applied the newly proposed snpboost algorithm on large-scale genotype data of the UKBB. In particular, we have compared the performance of snpboost to the one achieved by the lasso *via* snpnet, which has been shown to outperform many classical PRS (Qian et al., 2020). Our results indicate that the snpboost algorithm leads to PRS models that are highly competitive to lasso-based PRS models in both predictive performance and computation time. Although it might have been expected that the computation time would be higher for statistical boosting than for the lasso (Hepp et al., 2016), our approach had a tendency to result in sparser models. These sparser models correspond to an earlier stopping of the algorithm which reduces the computation time of boosting. The incorporation of further covariates such as age, sex and principal components in the fitting process of the PRS resulted in increased computation times for some phenotypes, particularly for height. However, in such cases, the boosting algorithm yielded an improved predictive performance with larger numbers of included variants. This illustrates that sparsity is not always favorable in regards of predictive performance. Additionally, we compared the performance of snpboost to further predictive PRS tools, which are either summary statistics based as PRScs, LDpred2 and SBayesR or multivariable approaches *via* the LDAK implementation of BayesR, Ridge Regression and Bolt-LMM (Zhang et al., 2021). While these methods do not apply variable selection, the predictive performance of snpboost was still highly competitive.

Our analyses show that there is a large overlap of the chosen variants by lasso and boosting, in particular regarding the variants with high estimated effect sizes. However, boosting has been found to include less variants in the final model and to induce less shrinkage on the effect estimates compared to the lasso. In clinical practice, a sparser PRS model might be of particular interest if the aim is not only prediction but also the identification of risk loci in the genome. In fact, functional annotations of the selected variants can better elucidate the underlying biological mechanisms influencing the analyzed trait. Thus, statistical boosting might be one way to yield more biologically interpretable PRS models.

Despite the presented promising results, the proposed method also inherited some limitations from statistical boosting. In contrast to classical regression methods, boosting does not provide closed formulas for standard errors of effect estimates or confidence intervals that could be used for inference. Furthermore, as mentioned before, statistical boosting is in general associated with a slightly higher computational complexity compared to methods such as the lasso (Hepp et al., 2016) and has a known tendency to include too many variables in low-dimensional settings (Staerk and Mayr, 2021; Strömer et al., 2022). Our results suggest that the incorporation of batches substantially reduced the computational time. Additionally, the reduction of the search space in each boosting step might partially prevent the algorithm from selecting too many variables. However, the implementation of the batch-based statistical boosting in snpboost is currently limited to linear base-learners, each corresponding to one genetic variant.

Apart from those technical limitations, using individual-level data raises ethical and logistical questions: While summary statistics are easily shared and do not allow for identification of unique individuals, individual-level data involve the risk of identification. It is therefore crucial, that providers as well as researchers using individual-level data follow ethical standards. Furthermore, the storage and transfer of individual-level data require more capacities which might not be at everyone’s disposal in the complete research community. However, the resulting PRS can be published by sharing only the included variants, alleles and coefficients—exactly like summary statistics (Lambert et al., 2021). To make use of available summary statistics and to avoid the limitations associated with individual-level data, it might be of interest to develop an approximation of a component-wise boosting algorithm based on summary statistics and LD panels, analogously as lassosum for the lasso. From a computational perspective, this is not necessary as snpboost only requires limited resources (e.g., our analysis of the UKBB data was run with only 32 GB RAM in total).

By incorporating the log loss we made our framework applicable also to binary traits and demonstrated the convenience of further extensions of the snpboost framework beyond the case of continuous phenotypes. Without re-specifying our hyperparameters we were able to yield similar results as the snpnet framework.

In future research we want to further exploit the modular structure of boosting to model more complex biological phenomena. We will incorporate different loss functions to extend the snpboost framework to be applicable also to count and time-to-event data. To account for the uncertainty in the prediction, one could also construct subject-specific prediction intervals based on quantile regression (Mayr et al., 2012). Besides extending the approach *via* new loss functions, one could also change the base-learners in various ways. For example, base-learners could be adapted to take into account different models of inheritance beside the classical additive component typically used in the polygenic models, such as dominant and recessive hereditary schemes. Further possibilities for future research include the extension of the set of possible base-learners, e.g., to model gene-environment interactions as well as epistatic effects across variants which can play a relevant role in biological phenotypes (Li and Lehner, 2020). To do so, base-learners including interactions between variants and variant-covariate interactions could be incorporated. Apart from that, biological knowledge can also be used a priori. Márquez-Luna et al. (2021) have shown that the incorporation of functional annotations of the genetic variants contribute to a rise in prediction accuracy. Previous works in the field of penalized regression and boosting have proposed to make use of biologically meaningful groups of genomic variants such as genes or pathways as described by Luan and Li (2008), Wei and Li (2007) as well as Liu et al. (2013). While those previous methods were computationally limited to smaller datasets our framework opens the possibility to include those ideas in the multivariable modelling of PRS. Besides those methodological extensions of our proposed snpboost framework, future research will also focus on the practical application of PRS derived by our framework. An important aspect of PRS research is the transferability of PRS models to different ethnicities, as PRS are often derived on cohorts of European ancestry and a substantial loss of predictive performance is observed when applied on further cohorts with different ethnicities (Landry et al., 2018; Evans et al., 2022). Previous studies have indicated that sparser models may contribute to overcome this issue (Maj et al., 2022) and it is of particular interest to examine the transferability of PRS derived by statistical boosting.

## Data Availability

The data analyzed in this study is subject to the following licenses/restrictions: This research has been conducted using the UK Biobank resource under application number 81202 (http://www.ukbiobank.ac.uk). Requests to access these datasets should be directed to UK Biobank, http://www.ukbiobank.ac.uk.
