# Supplementary material for: A statistical boosting framework for polygenic risk scores based on large-scale genotype data
Source: Front Genet. 2023 Jan 10;13:1076440. doi: 10.3389/fgene.2022.1076440 (PMC9871367; doi:10.3389/fgene.2022.1076440)
Supplement: Supplementary file 1 [file DataSheet1.pdf]

frontiers

Supplementary Material

SIMULATION STUDY

Simulations are based on the UK Biobank genotype data (Bycroft et al., 2018) obtained under application number 81202 combined with simulated phenotypes. We restricted the individuals to white British ancestry and used the PLINK 2.0 function `--thin-indiv-count` to randomly sample n individuals, of which 50%, 20% and 30% were assigned to the training, validation and test set, respectively (Chang et al., 2015; Purcell and Chang, 2015). Then, p variants with minor allele frequency not less than 1 % were randomly sampled using PLINK 2.0's `--thin-count`. Missing genotypes were replaced by the reference allele using the R package `bigsnpr` (Privé et al., 2018). Continuous phenotypes were simulated from a linear model with Gaussian distributed noise and effect sizes using `bigsnpr`. For each setting of h^2 and s, we simulated 100 different datasets. PRS models were derived by snpboost and evaluated by using various metrics regarding the predictive performance and the accuracy of the estimated coefficients. Furthermore, snpnet was applied on the simulated data using the default values (stopping lag 10, batch size 1,000).

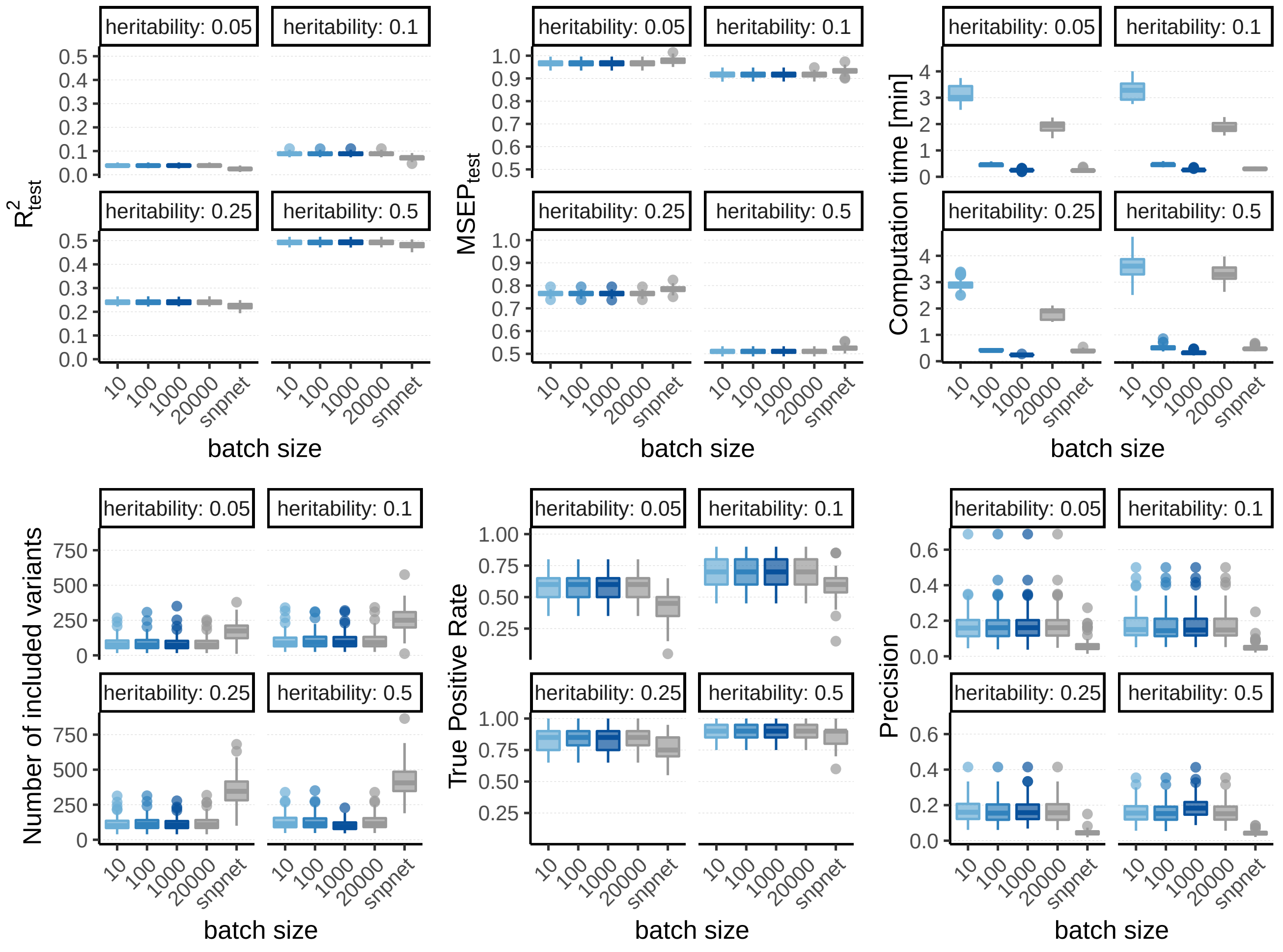

Figure S1. Results of 100 simulated phenotypes with varying heritability and sparsity $s = 0.1\%$ for $p = 20,000$ variants and $n = 20,000$ individuals (divided into 50% training, 20 % validation and 30% test set). Boxplots of the evaluation metrics obtained after $1,500$ boosting iterations are shown depending on the batch size. Batch size $p_{\text{batch}} = 20,000$ corresponds to the original L_2-boosting (shown in grey).

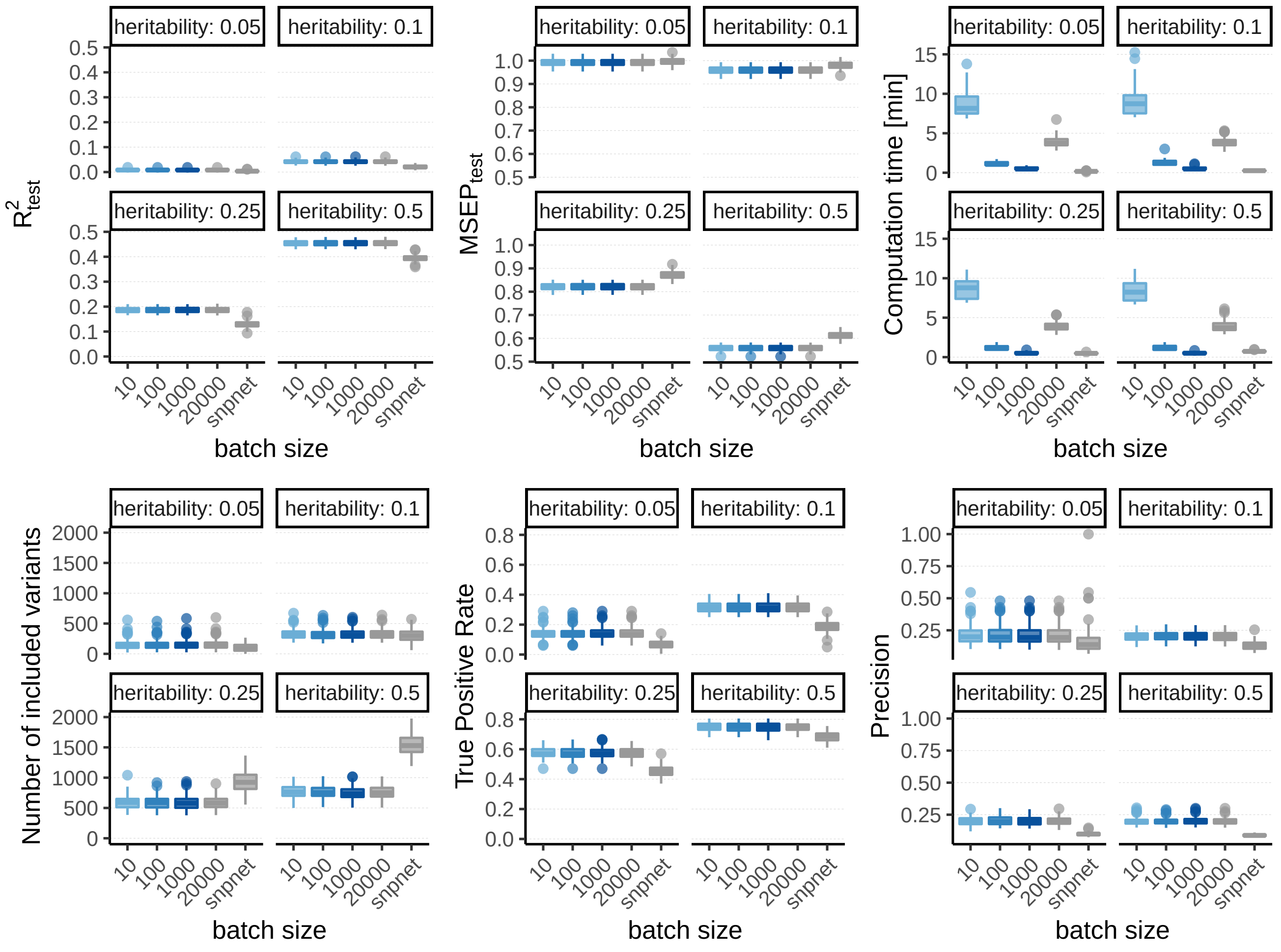

Figure S2. Results of 100 simulated phenotypes with varying heritability and sparsity $s = 1\%$ for $p = 20,000$ variants and $n = 20,000$ individuals (divided into 50% training, 20 % validation and 30% test set). Boxplots of the evaluation metrics obtained after $3,500$ boosting iterations are shown depending on the batch size. Batch size $p_{\text{batch}} = 20,000$ corresponds to the original L_2-boosting (shown in grey).

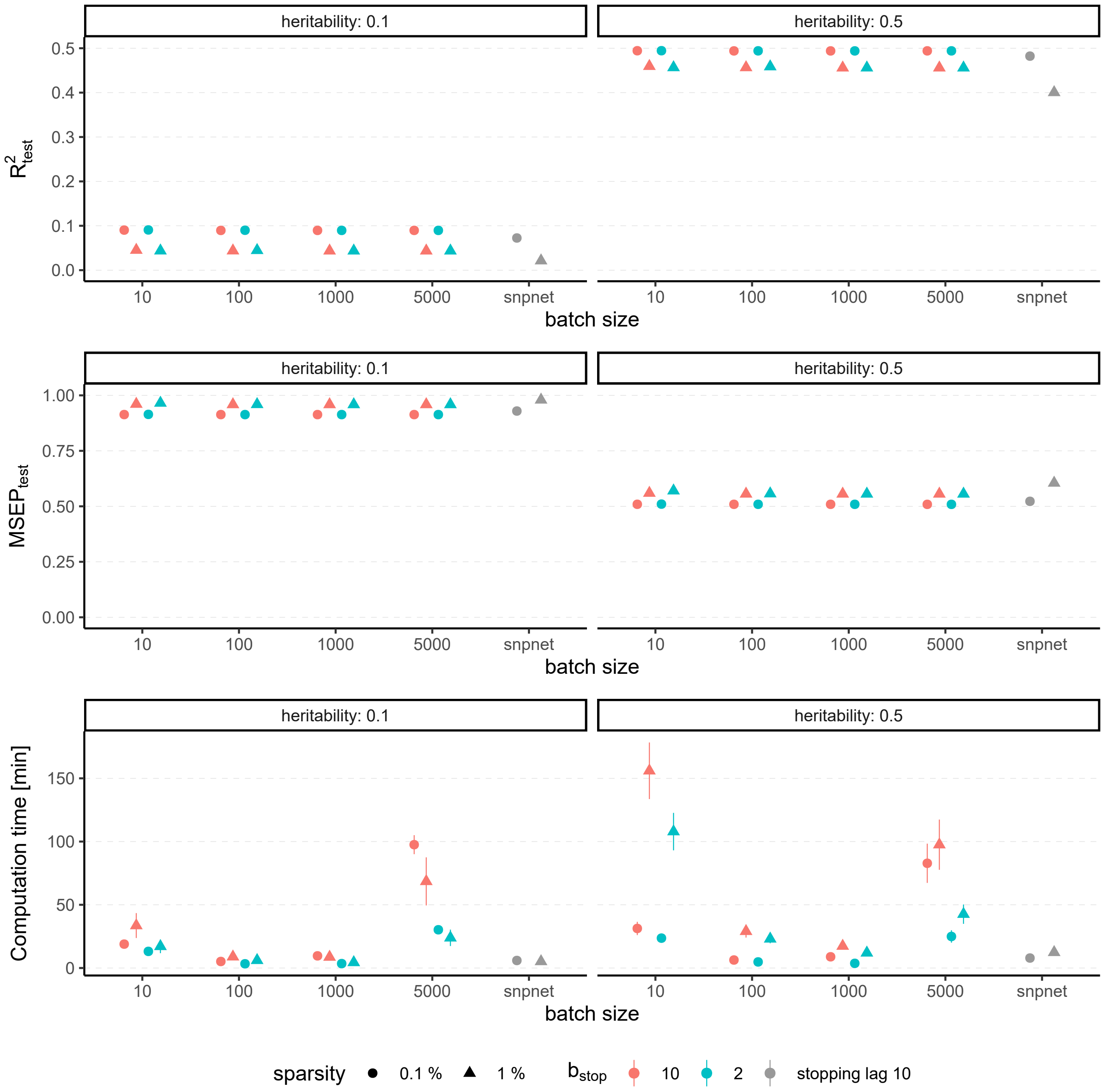

Figure S3. Results of 100 simulated phenotypes with heritability $h^2 \in \{10\%, 50\%\}$, sparsity $s \in \{0.1\%, 1\%\}$ and $b_{stop} \in \{2, 10\}$ for $p = 100,000$ variants and $n = 100,000$ individuals (divided into 50% training, 20 % validation and 30% test set). Mean and standard deviation of the evaluation metrics are shown depending on the batch size.

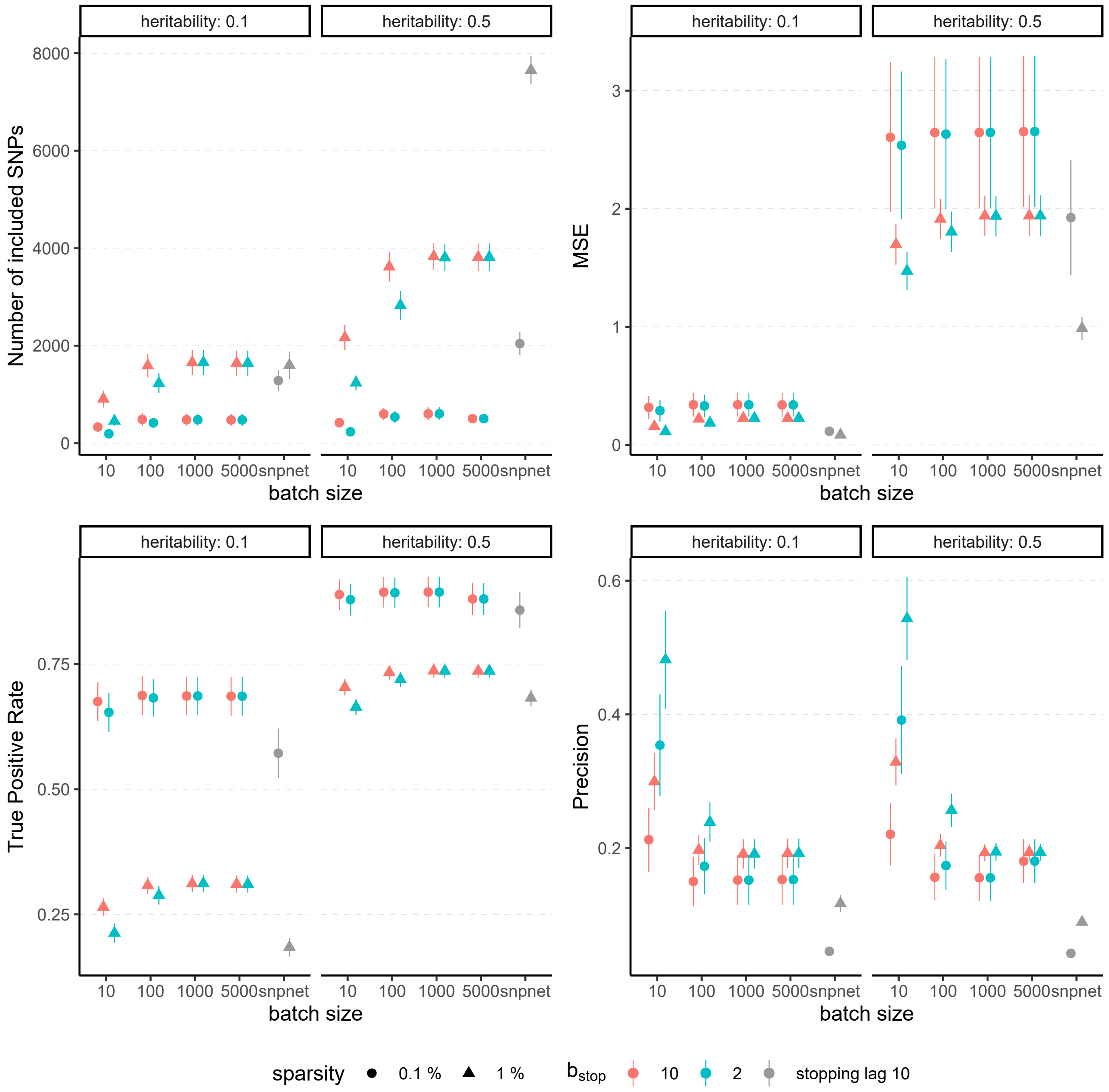

Figure S4. Evaluation metrics of the estimated coefficients for 100 simulated phenotypes with heritability $h^2 \in \{10\%, 50\%\}$, sparsity $s \in \{0.1\%, 1\%\}$ and $b_{\text{stop}} \in \{2, 10\}$ for $p = 100,000$ variants and $n = 100,000$ individuals (divided into 50% training, 20 % validation and 30% test set). Mean and standard deviation of the evaluation metrics are shown depending on the batch size.

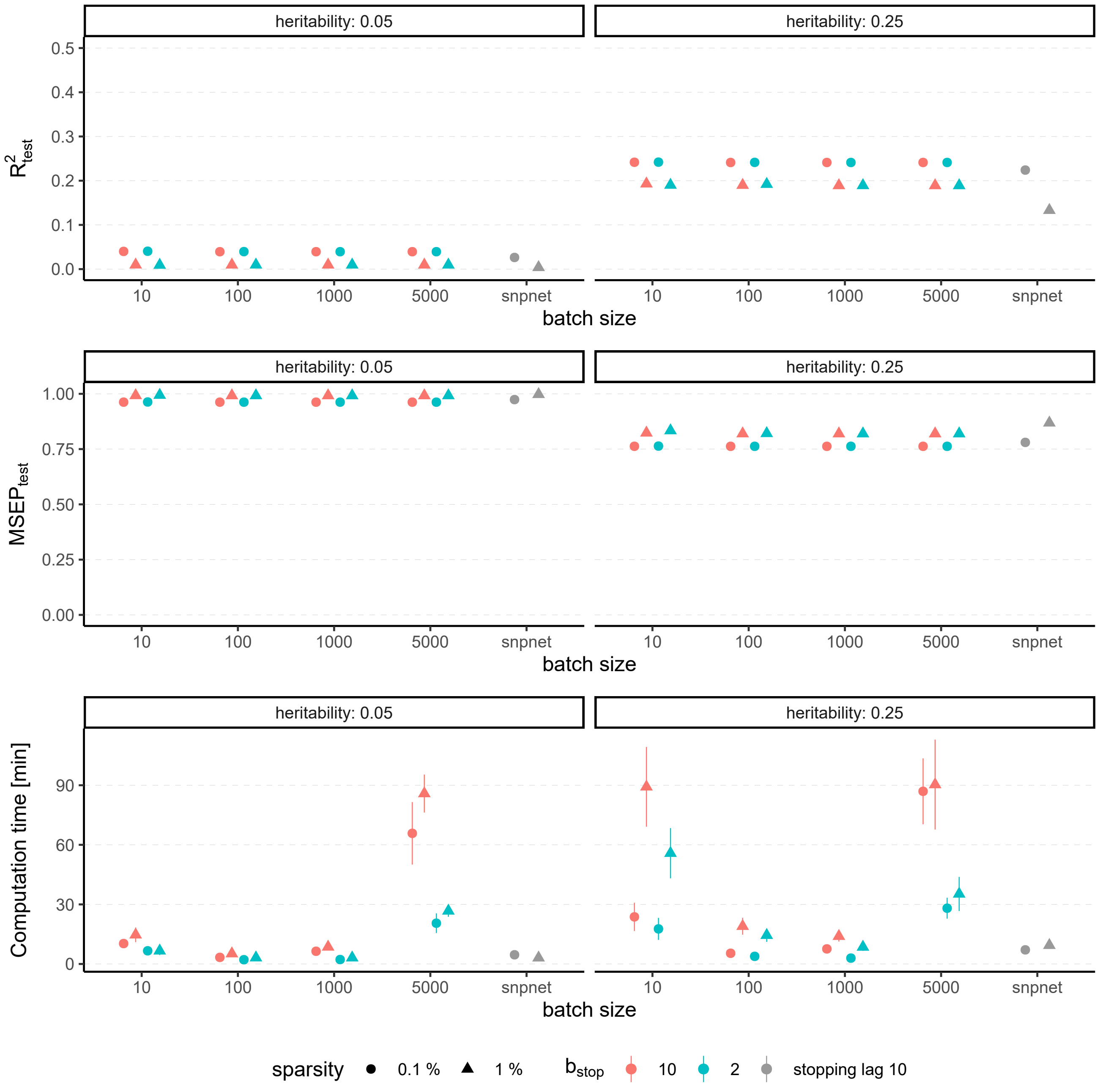

Figure S5. Results of 100 simulated phenotypes with heritability $h^2 \in \{5\%, 25\%\}$, sparsity $s \in \{0.1\%, 1\%\}$ and $b_{\text{stop}} \in \{2, 10\}$ for $p = 100,000$ variants and $n = 100,000$ individuals (divided into 50% training, 20 % validation and 30% test set). Mean and standard deviation of the evaluation metrics are shown depending on the batch size.

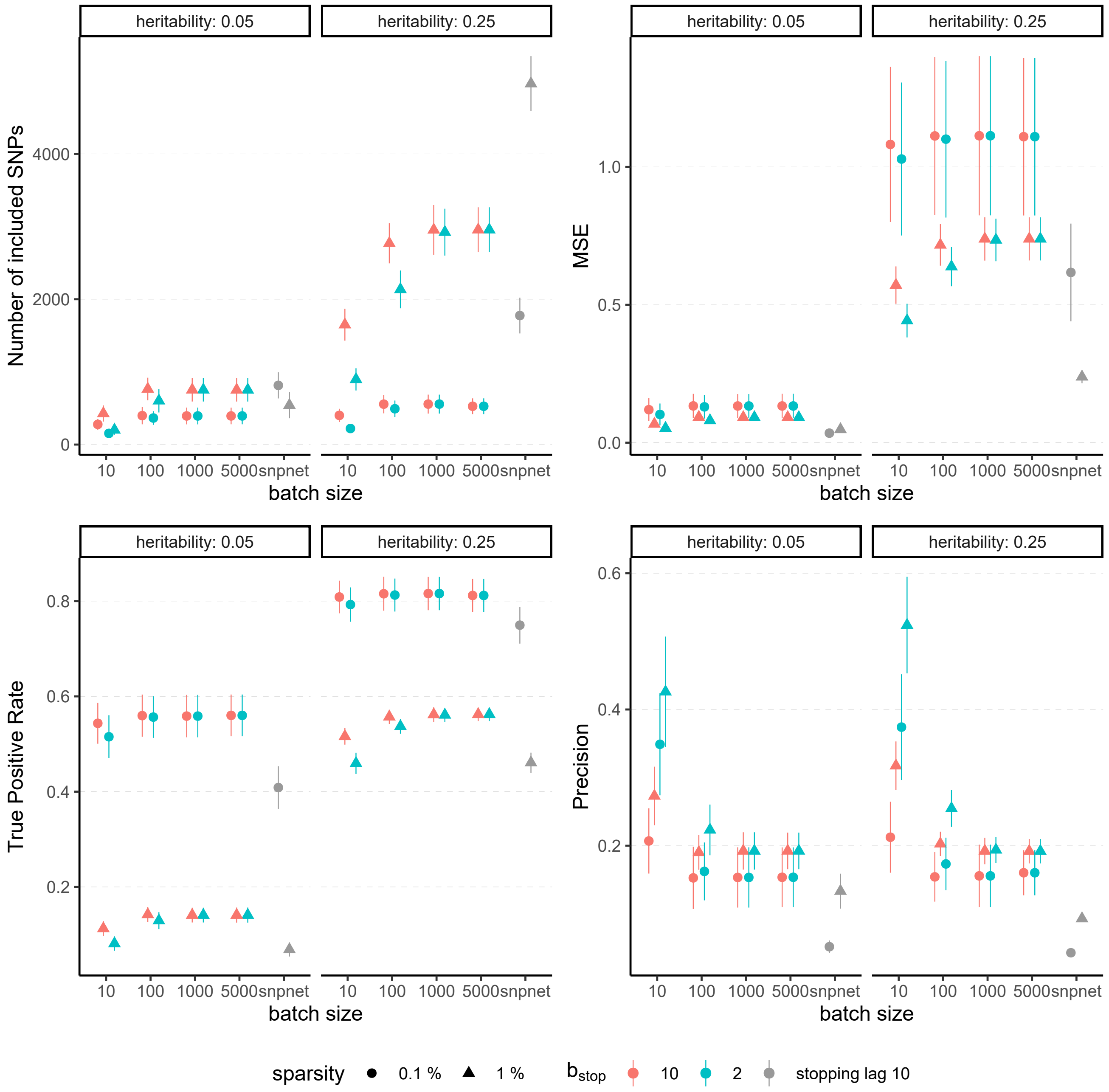

Figure S6. Evaluation metrics of the estimated coefficients for 100 simulated phenotypes with heritability $h^2 \in \{5\%, 25\%\}$, sparsity $s \in \{0.1\%, 1\%\}$ and $b_{\text{stop}} \in \{2, 10\}$ for $p = 100,000$ variants and $n = 100,000$ individuals (divided into 50% training, 20 % validation and 30% test set). Mean and standard deviation of the evaluation metrics are shown depending on the batch size.

APPLICATION TO THE UK BIOBANK

Out of the over 500,000 individuals from UK Biobank (Bycroft et al., 2018) we filtered for individuals with self-reported white British ancestry (UKBB field 21000) and available data for all chosen phenotypes, resulting in $n = 284,342$ observations. Additionally, the covariates age and sex as well as the first ten principal components of the genotype matrix are available. We randomly divided the data set into training ($n_{train} = 170,557$), validation ($n_{val} = 56,801$) and test set ($n_{test} = 56,984$). We used genome-wide genotype data and filtered for variants with a genotyped rate of at least 90% and a minor allele frequency of at least 0.1%, resulting in $p = 562,684$ genetic variants. Missing genotypes are imputed by the corresponding mean of the complete observations.
For both the boosting and lasso approaches, we first estimated a PRS using only the genotyped variants as predictors. We used the training set to fit the model and the validation set to simultaneously monitor the predictive performance for choosing the main tuning parameters of the algorithms (i.e., the number of iterations for boosting and the penalty parameter for the lasso). To fit the lasso we used the R package `snpnet` (Qian et al., 2020) with the provided default hyperparameters. Following the results of our simulation study, for the snpboost algorithm we chose a batch size of $p_{\text{batch}} = 1,000$ variants, a learning rate of $\nu = 0.1$ and an outer stopping lag of $b_{\text{stop}} = 2$ batches.

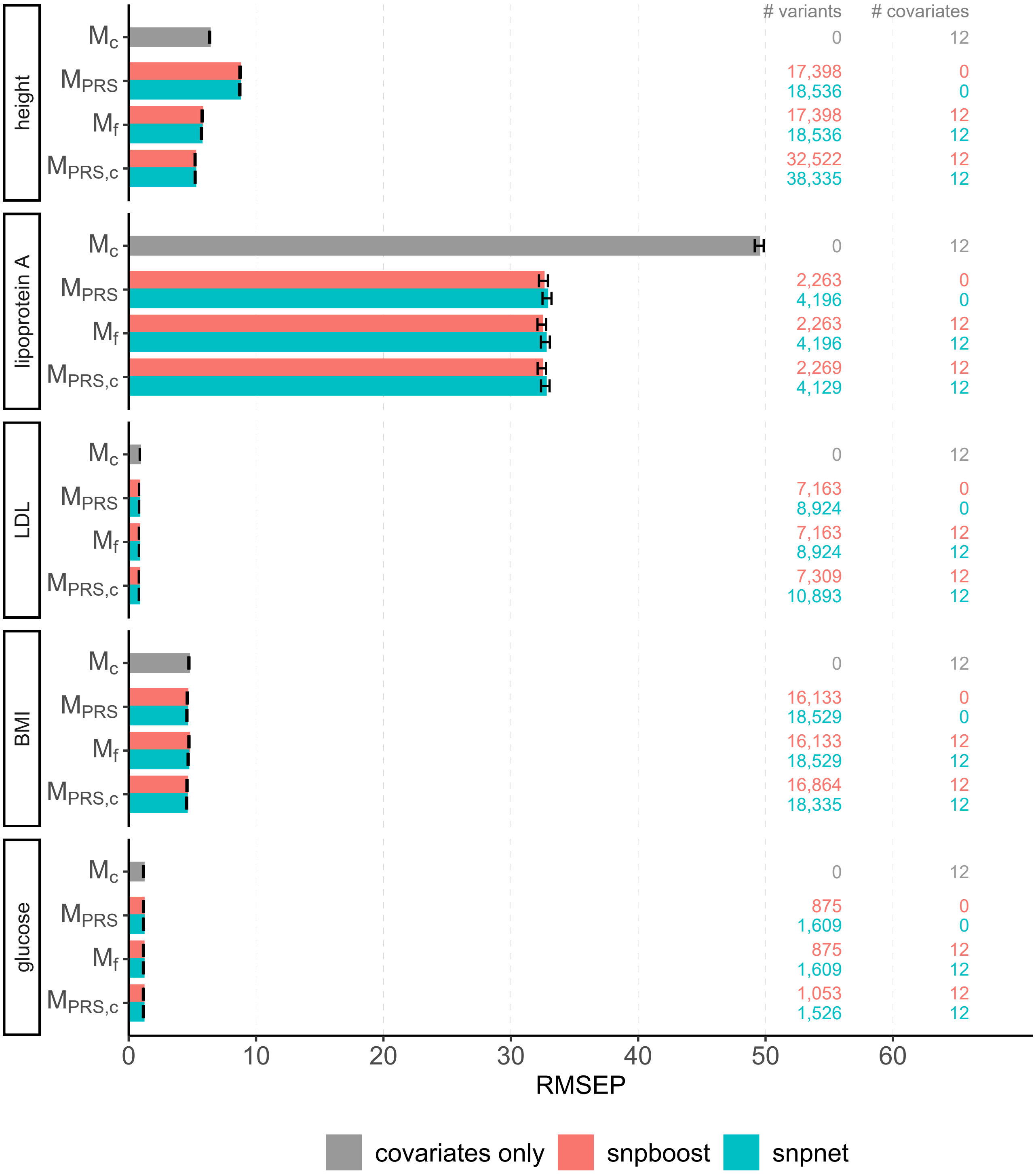

Figure S7. Comparison of predictive performance of snpnet and snpboost for five continuous phenotypes from the UKBB. Results of the covariate-only model (M_c, grey bars) and multivariable polygenic models with and without inclusion of the covariates derived by lasso (snpnet, petrol-coloured bars) and statistical boosting (snpboost, red-coloured bars) for the prediction of five phenotypes from the UKBB. The barplots show the predictive performance ($RMSEP$) on the test set of 52,551 unrelated white British individuals. M_{PRS} corresponds to a linear model incorporating the PRS as a single predictor variable and M_f to a linear model incorporation sex, age and the first ten principal components as additional covariates. $M_{\text{PRS},c}$ includes the covariates already in the fitting process of the PRS. Bootstrapped 95% confidence intervals are indicated by error bars. Furthermore, information on the number of selected genetic variants (# variants) and the number of additionally included covariates (# covariates) is given.

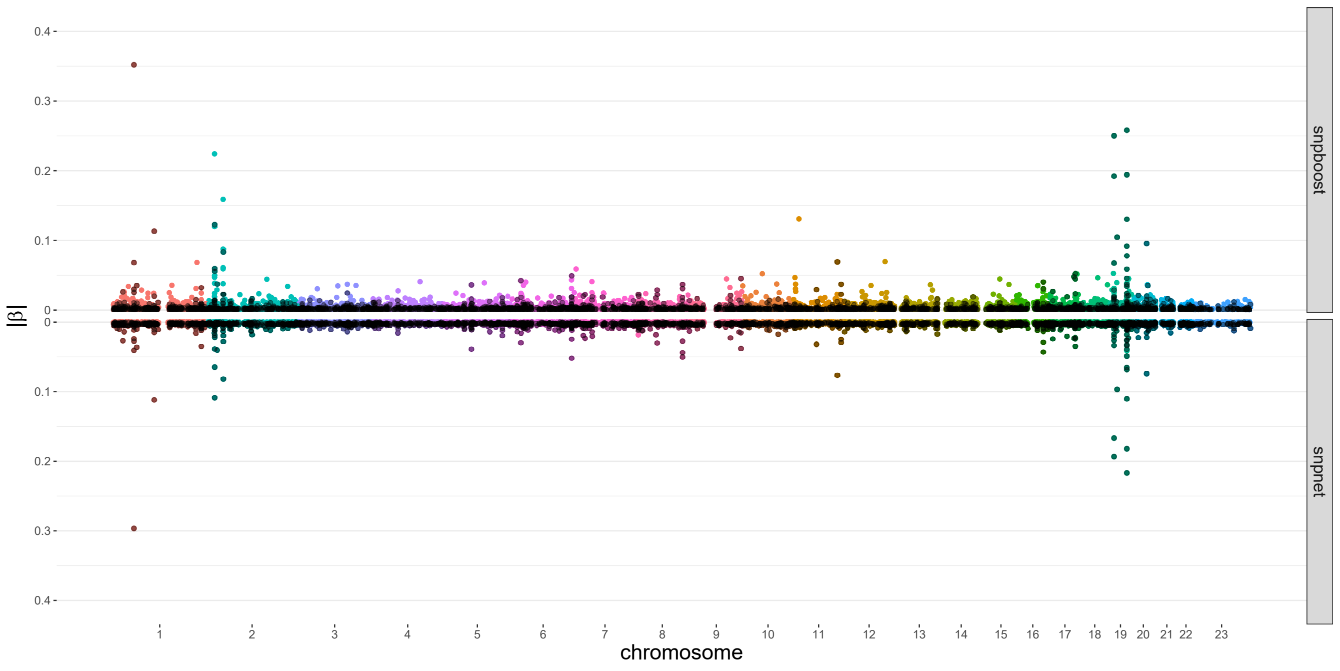

Figure S8. Absolute values of coefficient estimates for PRS models for LDL-cholesterol derived by boosting (snpboost) and lasso (snpnet) shown in dependence of the genomic position of the variants. Variants that are included in both models are marked in black.

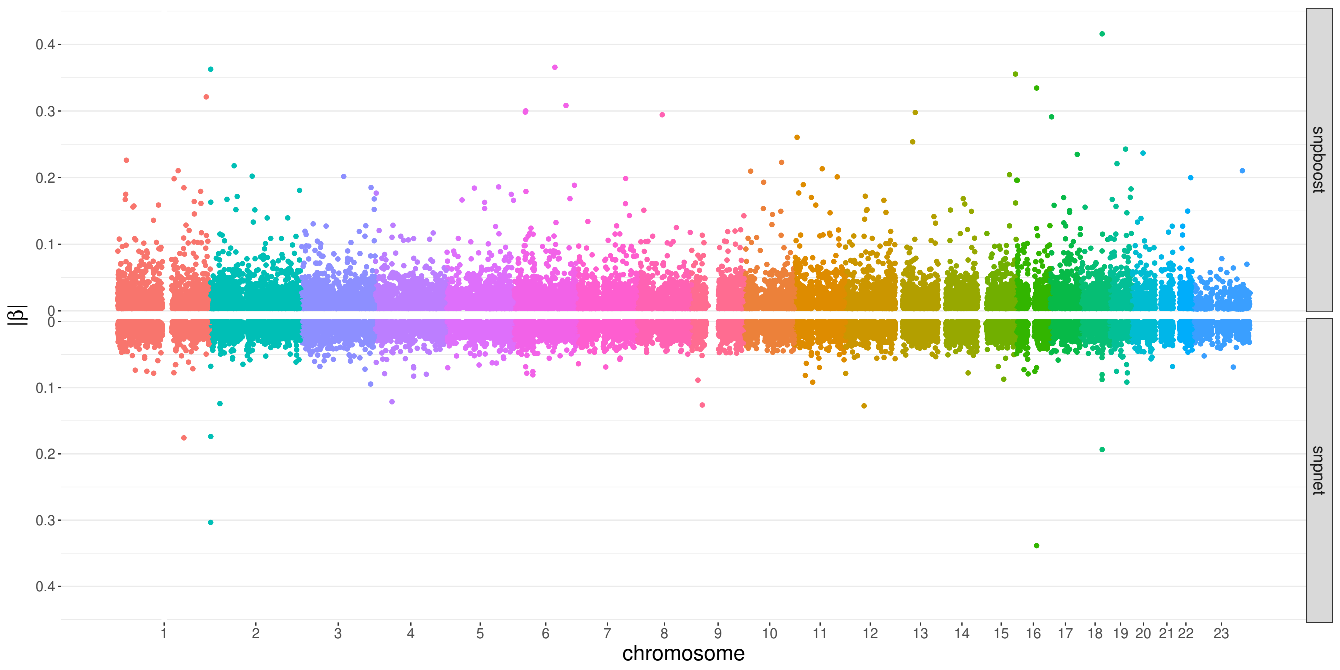

Figure S9. Absolute values of coefficient estimates for PRS models for BMI derived by boosting (snpboost) and lasso (snpnet) shown in dependence of the genomic position of the variants.

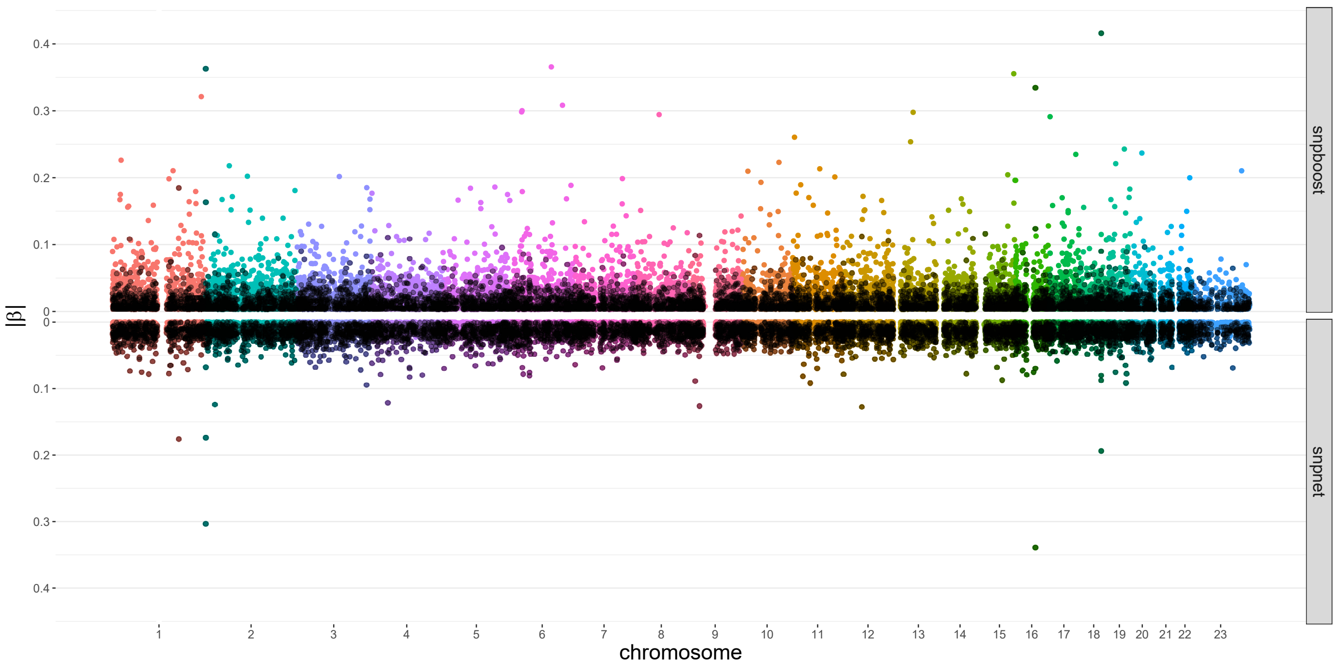

Figure S10. Absolute values of coefficient estimates for PRS models for BMI derived by boosting (snpboost) and lasso (snpnet) shown in dependence of the genomic position of the variants. Variants that are included in both models are marked in black.

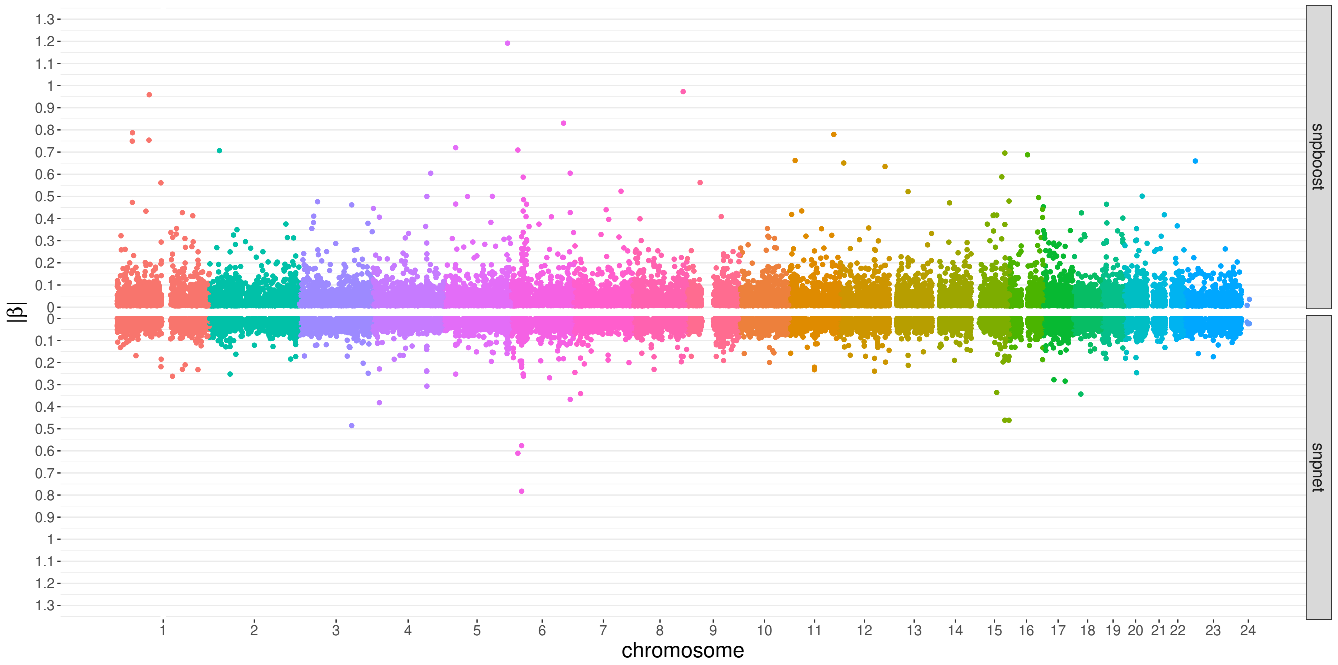

Figure S11. Absolute values of coefficient estimates for PRS models for height derived by boosting (snpboost) and lasso (snpnet) shown in dependence of the genomic position of the variants.

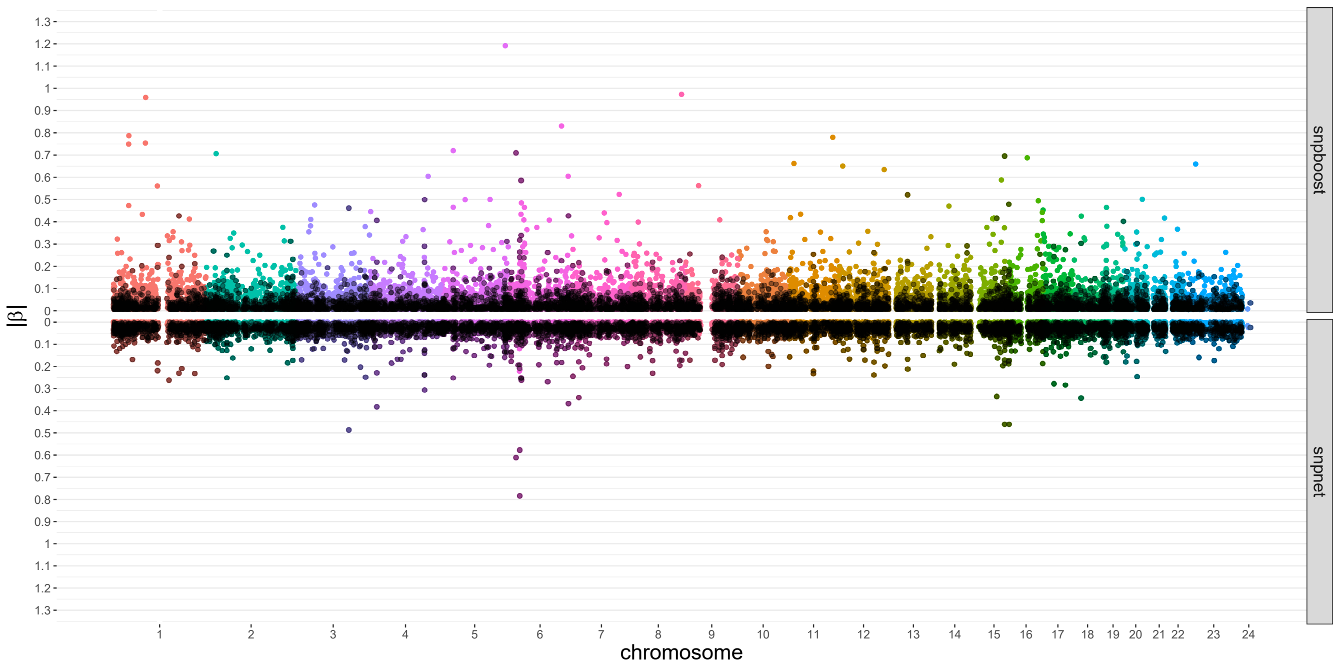

Figure S12. Absolute values of coefficient estimates for PRS models for height derived by boosting (snpboost) and lasso (snpnet) shown in dependence of the genomic position of the variants. Variants that are included in both models are marked in black.

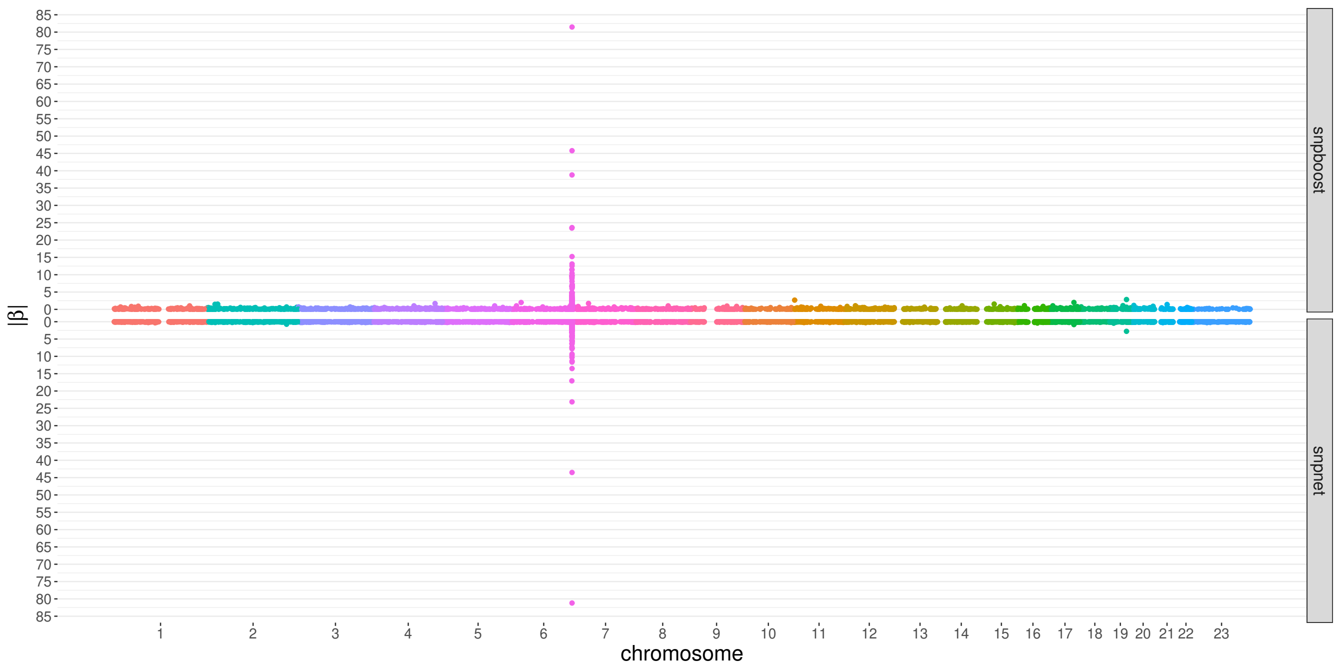

Figure S13. Absolute values of coefficient estimates for PRS models for lipoprotein A derived by boosting (snpboost) and lasso (snpnet) shown in dependence of the genomic position of the variants.

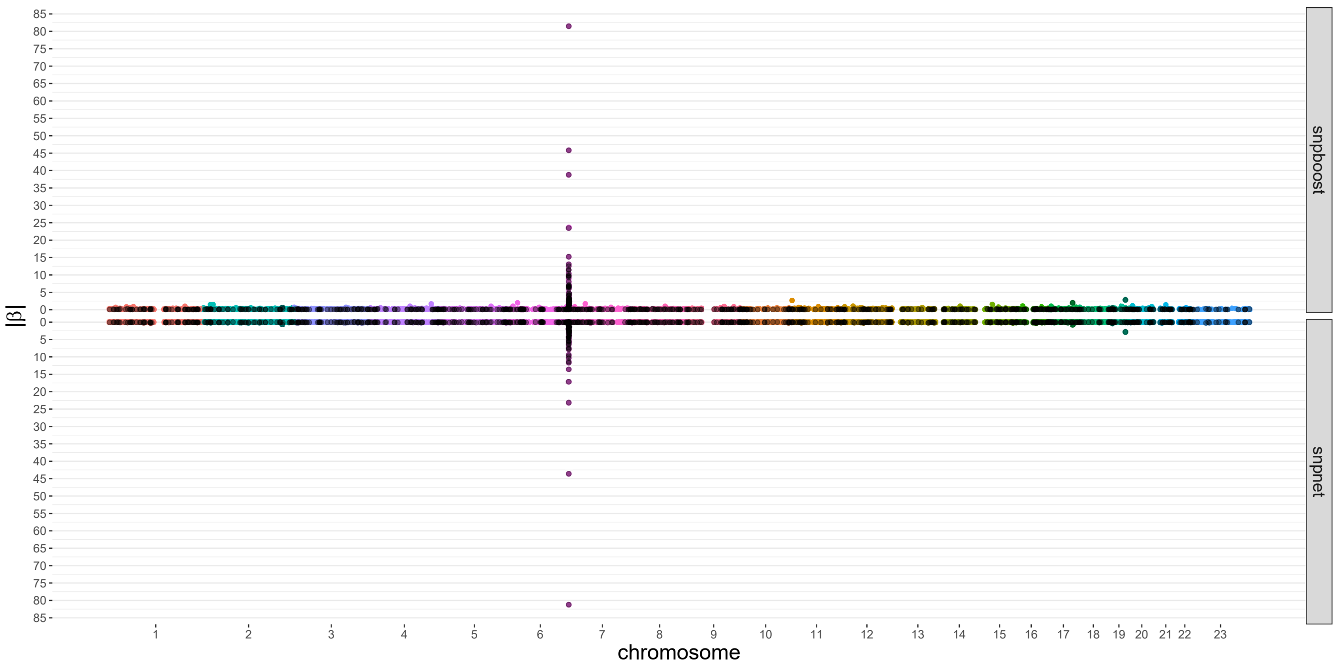

Figure S14. Absolute values of coefficient estimates for PRS models for lipoprotein A derived by boosting (snpboost) and lasso (snpnet) shown in dependence of the genomic position of the variants. Variants that are included in both models are marked in black.

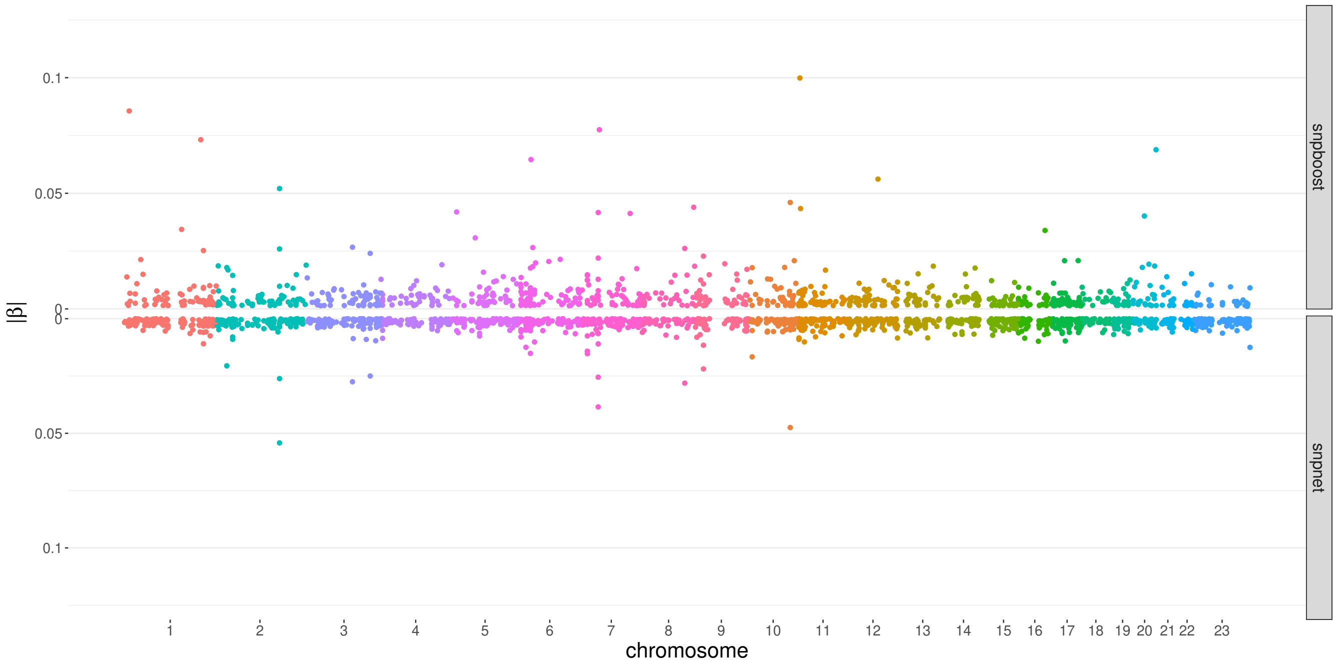

Figure S15. Absolute values of coefficient estimates for PRS models for glucose derived by boosting (snpboost) and lasso (snpnet) shown in dependence of the genomic position of the variants.

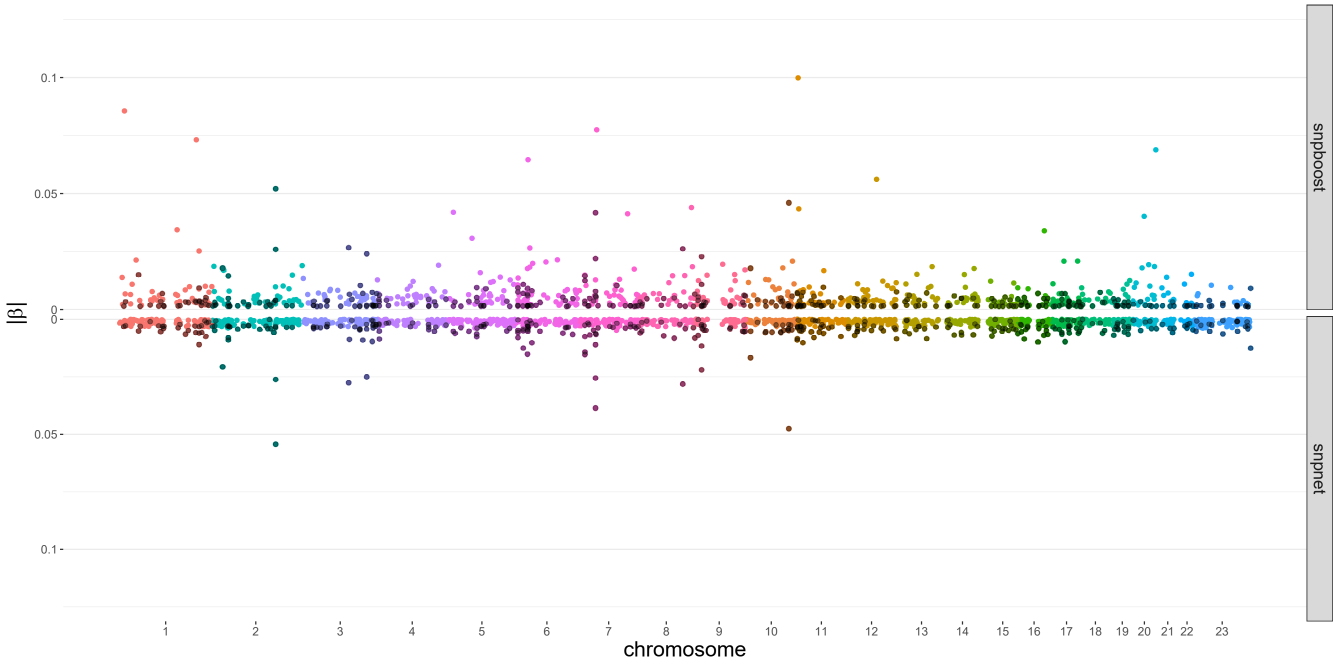

Figure S16. Absolute values of coefficient estimates for PRS models for glucose derived by boosting (snpboost) and lasso (snpnet) shown in dependence of the genomic position of the variants. Variants that are included in both models are marked in black.

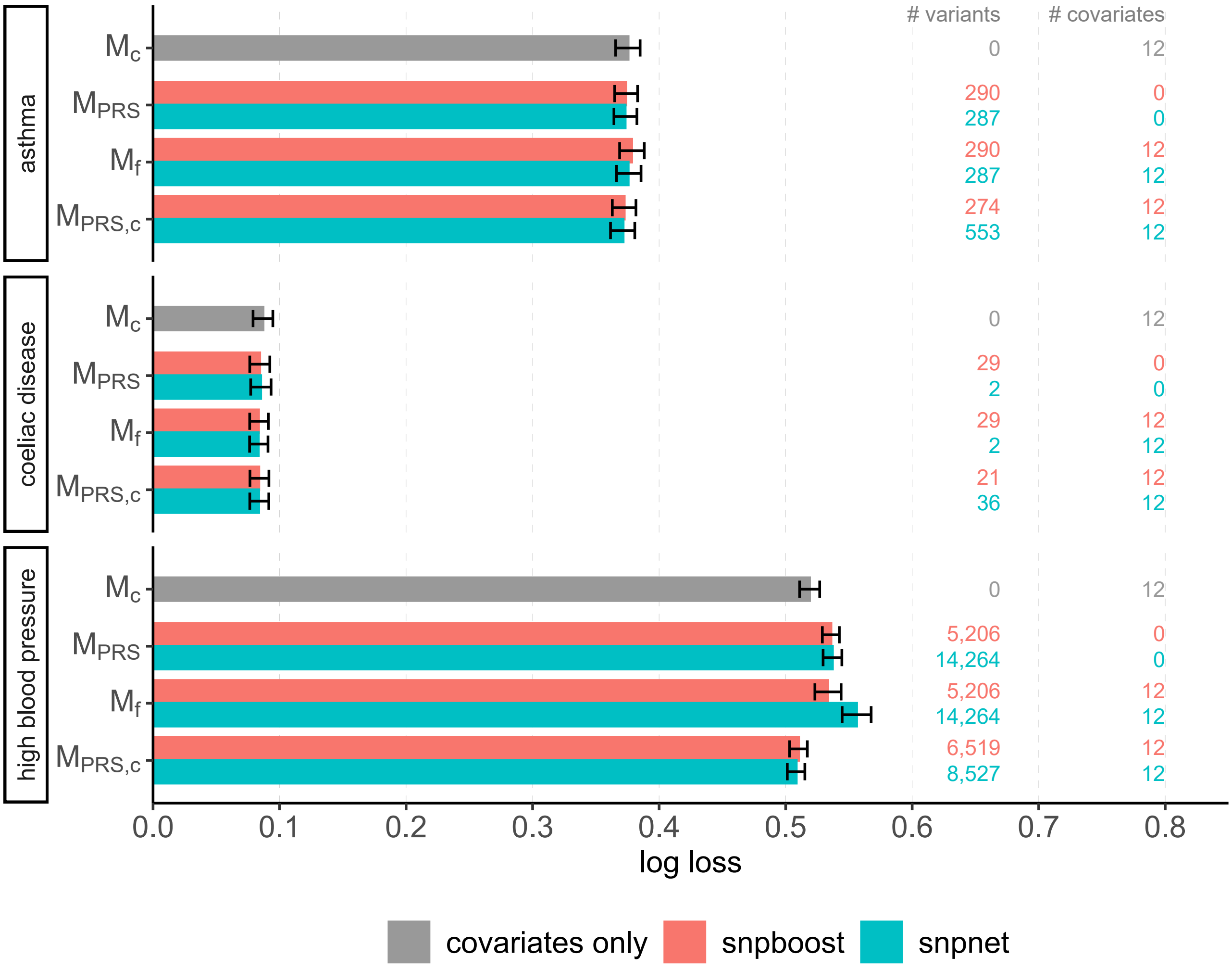

Figure S17. Comparison of predictive performance of snpnet and snpboost for three binary phenotypes from the UKBB. Results of the covariate-only model (M_c, grey bars) and multivariable polygenic models with and without inclusion of the covariates derived by lasso (snpnet, petrol-coloured bars) and statistical boosting (snpboost, red-coloured bars) for the prediction of five phenotypes from the UKBB. The barplots show the log loss on the test set of 20,164 (asthma), 28,513 (coeliac disease) and 78,741 (high blood pressure) unrelated white British individuals. M_{PRS} corresponds to a logistic regression model incorporating the PRS as a single predictor variable and M_f to a logistic regression model incorporation sex, age and the first ten principal components as additional covariates. $M_{\text{PRS},c}$ includes the covariates already in the fitting process of the PRS. Bootstrapped 95% confidence intervals are indicated by error bars. Furthermore, information on the number of selected genetic variants (# variants) and the number of additionally included covariates (# covariates) is given.

COMPARISON TO OTHER METHODS

To further validate our results, we compared snpboost based PRS for five continuous phenotypes (height, BMI, LDL cholesterol, lipoprotein A as well as glucose) from the UKBB to PRS derived by other methods besides snpnet. To make all chosen approaches applicable we restricted the dataset to unrelated white British individuals with all five phenotypes available and excluded sex chromosomes from the genotype data. We randomly divided the data set into training ($n_{train} = 157,204$), validation ($n_{val} = 52,416$) and test set ($n_{test} = 52,551$). We used genome-wide genotype data and filtered for variants with a genotyped rate of at least 90% and a minor allele frequency of at least 0.1% resulting in $p = 550,679$ genetic variants. First we compared snpboost to PRScs (Ge et al., 2019), which is a Bayesian approach that applies shrinkage on summary statistics based on a provided LD reference. To derive summary statistics we conducted a GWAS on the training set via plink2's `--glm`. We then used the validation set as well as the LD reference based on UKBB data provided at `https://github.com/getian107/PRScs` to run PRScs and derive the final coefficient estimates. As a second method based on summary statistics we applied LDpred2 (Privé et al., 2021) which is a Bayesian approach as well and incorporated a LD reference. Again, we used the summary statistics derived from the training set and estimated the LD matrix from our validation dataset according to the tutorial provided at `https://privefl.github.io/bigsnpr/articles/LDpred2.html`. We then fitted the LDpred2-inf model to retrieve the final estimates. Finally, we included SBayesR (Lloyd-Jones et al., 2019) in our comparison which is an extension of the individual-level data method BayesR (Moser et al., 2015) to summary statistics. We did run SBayesR by using the same summary statistics derived from our training data and the sparse LD matrix based on 50,000 unrelated individuals of European ancestry in the UKBB provided at `https://cnsgenomics.com/software/gctb/#Download`. For lipoprotein A we faced convergence issues for SBayesR. Following the authors' suggestions we excluded variants with a high p-value ($p > 0.2$, based on GWAS results) from the analysis, resulting in 24,102 variants.

Apart from PRScs, LDpred2 and SBayesR which all work on summary statistics we also included three approaches to fit multivariable models on genotype data via LDAK (version 5.2, `https://dougspeed.com/`, Speed et al. (2012)). All LDAK-Predict models (Zhang et al., 2021) are applied on individual-level genotype data. To do so a pre-predictor heritability matrix for each phenotype has to be constructed. Following the tutorial at `http://dougspeed.com/per-predictor-heritabilities/` we computed the pre-predictor heritabilities assuming the BLD-LDAK-model and using the GWAS results based our training set and the validation set to estimate the weights and heritabilities. Due to technical reasons only single-character alleles are processed. Furthermore, for each phenotype, variants with 0% predicted heritability are excluded from the analysis. Following this pre-processing step we fitted three LDAK-Predict models:
LDAK-Predict models are a generalized version of an existing Bayesian tool respectively (Zhang et al., 2021). They can be applied to individual-level-data and use the same priors as their corresponding existing tool for summary statistics. LDAK-Bolt-Predict uses the same two gaussian priors as Bolt-LMM (Loh et al., 2015) for each genetic variant. This is also true for the tools LDAK-BayesR-Predict and LDAK-Ridge-Predict which use the same priors as BayesR (Moser et al., 2015) and BLUP (best linear unbiased prediction, Henderson (1950)) respectively. While LDAK-Ridge-Predict uses a single gaussian prior per variant, LDAK-BayesR-Predict incorporates three gaussian priors and one point mass per variant (Zhang et al., 2021).
All LDAK methods were applied on the training set using the estimated pre-predictor heritabilities and the final model was chosen due to performance on the validation set. Resulting coefficients were used to derive

PRS via `--score` in plink2 (Chang et al., 2015; Purcell and Chang, 2015). Using the resulting $\hat{\text{PRS}}$ we then fitted two models on the training and validation sets, namely the first one (M_{PRS}) incorporating only the PRS as a single predictor variable:

$$M_{\text{PRS}} : Y = \gamma_0 + \gamma_{\text{PRS}}\widehat{\text{PRS}} \tag{S1}$$

and the second one (M_f) including the first ten principal components, sex and age as additional covariates:

$$M_f : Y = \gamma_0 + \gamma_{\text{PRS}}\widehat{\text{PRS}} + \gamma_1\text{PC}_1 + \cdots + \gamma_{10}\text{PC}_{10} + \gamma_{\text{sex}}\text{sex} + \gamma_{\text{age}}\text{age}. \tag{S2}$$

Resulting estimates were applied on the test set. Table S1 shows the prediction performance measured as R^2 of predicted and observed phenotype on the test set and table S2 shows the number of genetic variants with non-zero coefficient in the corresponding PRS model. Our proposed snpboost algorithm showed highly competitive performance in regards of the compared prediction tools. While PRScs, LDpred2-inf, SBayesR and the LDAK-Predict models do not induce sparsity, snpboost and snpnet yield sparse models without losing predictive accuracy.

Table S1. Comparison of predictive performance of eight PRS methods for five phenotypes from the UKBB. Results of GWAS-based (PRScs, LDpred2-inf and SBayesR) and individual-level data-based (Bolt, Ridge, BayesR, snpnet and snpboost) polygenic models with and without inclusion of the covariates for the prediction of three phenotypes from the UKBB. The table gives the predictive performance (R^2) on the test set of 55,221 unrelated white British individuals. M_{PRS} corresponds to a logistic regression model incorporating the PRS as a single predictor variable and M_f to a logistic regression model incorporating sex, age and the first ten principal components as additional covariates.

method	height		BMI		LDL		glucose		lipoprotein A	
	M_{PRS}	M_f	M_{PRS}	M_f	M_{PRS}	M_f	M_{PRS}	M_f	M_{PRS}	M_f
based on summary statistics										
PRScs	0.0764	0.5995	0.0416	0.0482	0.1241	0.1506	0.0101	0.0200	0.1943	0.1953
LDpred2	0.0595	0.5850	0.0574	0.0668	0.0748	0.0966	0.0055	0.0100	0.1272	0.1297
SBayesR	0.0802	0.6089	0.0647	0.0737	0.1403	0.1664	0.0146	0.0237	0.1836	0.1864
based on individual-level data										
Bolt	0.1159	0.5976	0.0941	0.0987	0.1492	0.1736	0.0147	0.0218	0.5330	0.5356
Ridge	0.0946	0.5662	0.0880	0.0924	0.0794	0.0975	0.0073	0.0117	0.1804	0.1811
BayesR	0.1197	0.6020	0.0873	0.0914	0.1501	0.1747	0.0147	0.0216	0.5365	0.5392
snpnet	0.1083	0.6176	0.0736	0.0792	0.1505	0.1765	0.0125	0.0234	0.5606	0.5634
snpboost	0.1039	0.6125	0.0621	0.0671	0.1546	0.1802	0.0115	0.0220	0.5675	0.5703

Table S2. Number of selected variants of eight PRS methods for five phenotypes from the UKBB. Results of GWAS-based (PRScs, LDpred2 and SBayesR) and individual-level data-based (Bolt, Ridge, BayesR, snpnet and snpboost) polygenic models with and without inclusion of the covariates for the prediction of five phenotypes from the UKBB. The table gives the number of genetic variants that are included in the PRS.

method	height	BMI	LDL	glucose	lipoprotein A
based on summary statistics					
PRScs	117,203	117,203	117,203	117,203	117,203
LDpred2	114,702	114,702	114,702	114,702	114,702
SBayesR	117,391	117,391	117,391	117,391	24,102
based on individual-level data					
Bolt	546,011	546,011	386,725	546,009	546,011
Ridge	546,011	546,011	386,725	546,009	546,011
BayesR	546,011	546,011	386,725	546,009	546,011
snpnet	18,140	18,118	8,671	1,491	4,017
snpboost	15,393	15,822	7,527	925	2,858

REFERENCES

Bycroft, C., Freeman, C., Petkova, D., Band, G., Elliott, L. T., Sharp, K., et al. (2018). The UK Biobank resource with deep phenotyping and genomic data. *Nature* 562, 203–209. doi:10.1038/s41586-018-0579-z

Chang, C., Chow, C. C., Tellier, L. C., Vattikuti, S., Purcell, S. M., and Lee, J. J. (2015). Second-generation PLINK: rising to the challenge of larger and richer datasets. *GigaScience* 4. doi:10.1186/s13742-015-0047-8

Ge, T., Chen, C.-Y., Ni, Y., Feng, Y.-C. A., and Smoller, J. W. (2019). Polygenic prediction via bayesian regression and continuous shrinkage priors. *Nature Communications* 10. doi:10.1038/s41467-019-09718-5

Henderson, C. R. (1950). Estimation of genetic parameters. *Annals of Mathematics Studies* 21, 309–310

Lloyd-Jones, L. R., Zeng, J., Sidorenko, J., Yengo, L., Moser, G., Kemper, K. E., et al. (2019). Improved polygenic prediction by bayesian multiple regression on summary statistics. *Nature Communications* 10. doi:10.1038/s41467-019-12653-0

Loh, P.-R., Tucker, G., Bulik-Sullivan, B. K., Vilhjálmsson, B. J., Finucane, H. K., Salem, R. M., et al. (2015). Efficient bayesian mixed-model analysis increases association power in large cohorts. *Nature Genetics* 47, 284–290. doi:10.1038/ng.3190

Moser, G., Lee, S. H., Hayes, B. J., Goddard, M. E., Wray, N. R., and Visscher, P. M. (2015). Simultaneous discovery, estimation and prediction analysis of complex traits using a bayesian mixture model. *PLOS Genetics* 11, e1004969. doi:10.1371/journal.pgen.1004969

Privé, F., Arbel, J., and Vilhjálmsson, B. J. (2021). Ldpred2: better, faster, stronger. *Bioinformatics* 36, 5424–5431. doi:10.1093/bioinformatics/btaa1029

Privé, F., Aschard, H., Ziyatdinov, A., and Blum, M. G. B. (2018). Efficient analysis of large-scale genome-wide data with two R packages: bigstatsr and bigsnpr. *Bioinformatics* 34, 2781–2787. doi:10.1093/bioinformatics/bty185

Purcell, S. and Chang, C. (2015). Plink 2.0. `www.cog-genomics.org/plink/2.0/`

Qian, J., Tanigawa, Y., Du, W., Aguirre, M., Chang, C., Tibshirani, R., et al. (2020). A fast and scalable framework for large-scale and ultrahigh-dimensional sparse regression with application to the UK Biobank. *PLOS Genetics* 16. doi:10.1371/journal.pgen.1009141

Speed, D., Hemani, G., Johnson, M., and Balding, D. (2012). Improved heritability estimation from genome-wide snps. *The American Journal of Human Genetics* 91, 1011–1021. doi:10.1016/j.ajhg.2012.10.010

Zhang, Q., Privé, F., Vilhjálmsson, B., and Speed, D. (2021). Improved genetic prediction of complex traits from individual-level data or summary statistics. *Nature Communications* 12. doi:10.1038/s41467-021-24485-y
